# Supplementary material for: Sperm migration in the genital tract—In silico experiments identify key factors for reproductive success
Source: PLoS Comput Biol. 2021 Jul 15;17(7):e1009109. doi: 10.1371/journal.pcbi.1009109 (PMC8282070; doi:10.1371/journal.pcbi.1009109)
Supplement: S1 Text — It is structured in 6 Supplementary Notes (A: Reconstruction of the bovine female genital tract, B: Sperm movement, C: Immune system, D: Sperm persistence, E: Thigmotaxis aids transition through cervix and UTJ, F: Computational Execution). (PDF) [file pcbi.1009109.s001.pdf]

S1 Text of Sperm migration in the genital tract - *in silico* experiments identify key factors for reproductive success

Jorin Diemer[1], Jens Hahn[1], Björn Goldenbogen[1], Karin Müller[2], and Edda Klipp[1]

[1]Theoretical Biophysics, Humboldt-Universität zu Berlin
[2]Leibniz Institute for Zoo and Wildlife Research

Contents

A Reconstruction of the bovine female genital tract **2**
A1 Vagina . . . 2
A2 Cranial Vagina . . . 2
A3 Cervix . . . 3
A4 Uterine body . . . 3
A5 Uterine Horns . . . 3
A6 Uterotubal junction . . . 3
A7 Oviducts . . . 4
A8 Concatenation . . . 4

B Sperm movement **4**
B1 Thigmotaxis . . . 4
B2 Fluid Field and positive rheotaxis . . . 5

C Immune system **8**

D Sperm persistence **8**

E Thigmotaxis aids transition through cervix and UTJ **8**

F Computational Execution **8**

Supplementary Note A: Reconstruction of the bovine female genital tract

All compartments of the female genital tract are considered as connected tubes, each described with a 3D cylindrical or conical function. The different compartments are connected in z direction. All parameters necessary for the description of the female genital tract are given in S1 Table. S1 Figure shows the result of the described volume and cross-sections at compartment transitions and interesting z positions. How each of the compartments was described mathematically is described below.

A1. Vagina. The vagina is described as a simple cylinder with radius r_v, Eq S1. Length and radius for the bovine vagina were set to $l_v = 25\,\mathrm{cm}$ and $r_v = 2.5\,\mathrm{cm}$, respectively. The cylindrical function f_v for the vagina is given by:

$$f_v(x,y) = x^2 + y^2 - r_v^2. \tag{S1}$$

A2. Cranial Vagina. The Cranial vagina compartment mimics the transition from vagina to cervix. Vagina and cervix mainly differ in two ways. First, they have a different radius. Second, the cervix holds primary and secondary folds. Therefore, the cranial vagina compartment must changes in radius and introduces primary and secondary folds dependent on the z-axis. The respective set of equations reads as follows and is explained below:

$$r_{cv}(z) = \frac{r_c - r_v}{l_{cv}} \cdot (z - z_{cv}) + r_v \tag{S2}$$

$$d_{center}(x,y) = \sqrt{x^2 + y^2} \tag{S3}$$

$$d_r = d_{center} - r_{cv} \tag{S4}$$

$$A_{pf}^{max} = \frac{r_c}{f_A^{pf}} \tag{S5}$$

$$A_{pf} = \frac{z - z_{cv}}{l_{cv}} \cdot A_{pf}^{max} \tag{S6}$$

$$A_{sf} = \begin{cases} A_{sf}^{max} & \cdot \overbrace{\frac{k_A^{pf} \cdot A_{pf} - \|d_r\|}{k_A^{pf} \cdot A_{pf}}}^{F_{A_{pf}}} \cdot \frac{(z - z_{cv}) - l_{cv} \cdot k_A^{sf}}{l_{cv} - l_{cv} \cdot k_A^{sf}} \\ & \text{if } \|d_r\| < k_A^{pf} \cdot A_{pf} \text{and } (z - z_{cv}) > l_{cv} \cdot k_A^{sf} \\ 0, & \text{otherwise} \end{cases} \tag{S7}$$

$$\alpha = \arccos\left(\frac{x}{d_{center}}\right) \tag{S8}$$

$$f_{cv}(x,y,z) = x^2 + y^2 - \left[r_{cv} + A_{pf} \cos(\omega_{pf} \cdot \alpha) + A_{sf} \sin\left(\omega_{sf} \pi \left(\frac{d_r - A_{pf} \cdot k_A^{pf}}{A_{pf} \cdot k_A^{pf}} \right) \right) \right]^2. \tag{S9}$$

Eq S2 defines the change of the cranial vagina radius r_{cv} from the vaginal radius r_v to the cervical radius r_c over the cranial vagina length l_{cv}.

Eqs S3 and S4 describe the distance to the compartment center d_{center}, that is the z-axis, and the distance to the nearest point on the cranial vagina radius d_r, respectively. A_{pf} (Eq S6) defines the amplitude (or depth) of the primary folds, which increases linearly with increasing z. Eq S7 defines the amplitude/depth of the secondary folds A_{sf}. The occurrence of secondary folds is limited in two regards. First, they only occur if primary folds are sufficiently large (which depends on the z-axis), i.e. when the difference between the z-coordinate and z_{cv} (the compartment baseline) is larger than l_{cv} times a constant k_A^{sf}, then the secondary fold amplitude A_{sf} becomes larger than 0. Second, they only occur in the middle of the primary folds, i.e. if the distance d_r is smaller than the depth of the primary fold times a constant k_A^{pf} (see first fraction named $F_{A_{pf}}$ in Eq S7, depicted in S2 Figure). Here, k_A^{sf} and k_A^{pf} represent the fractions of cranial vagina length and primary folds regions in which secondary folds occur, respectively, while A_{sf}^{max} is the maximal depth of secondary folds. Eq S8 defines the angle towards the x - axis. Finally, Eq S9 summarizes the contributions for the whole compartment. The squared x and y terms show that the equation still originates from a cylindrical description. The cosine term introduces ω_{pf} primary folds with amplitude A_{pf}, while the sine term introduces ω_{sf} secondary folds with amplitude A_{sf}. ω_{sf} is scaled by π and an additional term which ranges between -2 and 0, describing the whole circle, resulting in ω_{sf} secondary folds per primary fold.

A3. Cervix. The cervix holds primary and secondary folds, but compared to the cranial vagina it has a constant radius. Secondary folds are again limited to regions where primary folds are sufficiently deep, see Eq S11. The entire cervix is described by Eq S12. Note that the distance to the radius is defined in the same manner as for the cranial vagina (compare Eqs S4 and S10), but that the radius used in the compartment equations (Eqs S9 and S12) is fixed (r_v). The equations for the description of the cervix read:

$$d_r = d_{center} - r_c \tag{S10}$$

$$A_{sf} = \begin{cases} A_{sf}^{max}\left(\frac{k_A^{pf} A_{pf}^{max} - \|d_r\|}{k_A^{pf} A_{pf}^{max}}\right) & \text{if } \|d_r\| < k_A^{pf} A_{pf}^{max} \\ 0, & \text{otherwise} \end{cases} \tag{S11}$$

$$f_c(x,y) = x^2 + y^2 - \left[r_c + A_{pf}^{max}\cos(\omega_{pf}\alpha) + A_{sf}\sin\left(\omega_{sf}\pi\frac{d_r - k_A^{pf} A_{pf}^{max}}{k_A^{pf} A_{pf}^{max}}\right)\right]^2. \tag{S12}$$

A4. Uterine body. Similar to the cervical vagina compartment, the uterine body compartment has a changing radius and a decreasing depth of primary and secondary folds to zero. The logic behind the equations is the same as for the cranial vagina compartment. Again, the distance to the radius is defined as before, Eq S14. Here, r_{ub} is the radius of the uterine body and r_{uh_1} the radius at the lower end of the uterine horns. l_{ub} and z_{ub} are the length and the z baseline of the uterine body, respectively. The respective equations are:

$$r_{ub}(z) = \frac{r_{uh\_1} - r_c}{l_{ub}} \cdot (z - z_{ub}) + r_c \tag{S13}$$

$$d_r = d_{center} - r_{ub} \tag{S14}$$

$$A_{pf} = \frac{l_{ub} - (z - z_{ub})}{l_{ub}} \cdot A_{pf}^{max} \tag{S15}$$

$$A_{sf} = \begin{cases} A_{sf}^{max} \cdot \frac{k_A^{pf} \cdot A_{pf} - \|d_r\|}{k_A^{pf} \cdot A_{pf}} \cdot \frac{l_{ub} \cdot k_A^{sf} - (z - z_{ub})}{l_{ub} \cdot k_A^{sf}} \\ \quad \text{if } \|d_r\| < k_A^{pf} \cdot A_{pf} \text{and } (z - z_{ub}) < l_{ub} \cdot k_A^{sf} \\ 0, \quad \text{otherwise} \end{cases} \tag{S16}$$

$$f_{ub}(x,y,z) = x^2 + y^2 - \left[r_{ub} + A_{pf}\cos(\omega_{pf} \cdot \alpha) + A_{sf}\sin\left(\omega_{sf}\pi\left(\frac{d_r - A_{pf} \cdot k_A^{pf}}{A_{pf} \cdot k_A^{pf}}\right)\right)\right]^2. \tag{S17}$$

A5. Uterine Horns. The two uterine horns are described by the following equations.

$$r_{uh} = \frac{r_{uh\_2} - r_{uh\_1}}{l_{uh}}(z - z_{uh}) + r_{uh\_1} \tag{S18}$$

$$r_{zdiv} = \frac{r_{uh\_2} - r_{uh\_1}}{l_{uh}} z_{div} + r_{uh\_1} \tag{S19}$$

$$f_{uh} = \begin{cases} x^2 + \left(y - \sqrt[4]{\frac{z - z_{uh}}{z_{div}}} r_{zdiv}\right)^2 - r_{uh}^2 & \text{if } y \geq 0 \\ x^2 + \left(y + \sqrt[4]{\frac{z - z_{uh}}{z_{div}}} r_{zdiv}\right)^2 - r_{uh}^2 & \text{if } y < 0 \end{cases}. \tag{S20}$$

l_{uh}, r_{uh} and z_{uh} are length, radius and z offset of the uterine horns, while r_{uh_2} is the radius at the upper end of the uterine horns. z_{div} is the z position (with respect to z_{uh}) at which the two horns separate and r_{zdiv} is the radius of the uterine horns at this height. Eq S20 defines the shape of the uterine horns, one for $y \geq 0$ and one for $y < 0$. They have a decreasing radius (Eq S18) and two centers, which drift apart in the y direction.

A6. Uterotubal junction. In the uterotubal junction (UTJ) the radius decreases again, while the centers don't drift apart anymore, staying at a constant y position. The transition between the two compartments mimics the narrowest part of the whole tract, with radius r_{o_1}. The equations for the UTJ read as follows:

$$r_{utj} = \frac{r_{o\_1} - r_{uh\_2}}{l_{utj}}(z - z_{utj}) + r_{uh\_2} \quad \textbf{(S21)}$$

$$f_{utj} = \begin{cases} x^2 + \left(y - \sqrt[4]{\frac{l_{uh}}{z_{div}}} r_{zdiv}\right)^2 - r_{utj}^2 & \text{if } y \geq 0 \\ x^2 + \left(y + \sqrt[4]{\frac{l_{uh}}{z_{div}}} r_{zdiv}\right)^2 - r_{utj}^2 & \text{if } y < 0 \end{cases} . \quad \textbf{(S22)}$$

A7. Oviducts. Similar to the uterotubal junction the oviduct compartment only changes the radius of the two parallel tubes (Eq S23). The end of the oviduct has the radius r_{o_2}. In our simulations this height is never reached, as the question under investigation was when the sperms reach the oviductal compartment. The equations for the oviducts read:

$$r_o = \frac{r_{o\_2} - r_{o\_1}}{l_o}(z - z_o) + r_{o\_1} \quad \textbf{(S23)}$$

$$f_o = \begin{cases} x^2 + \left(y - \sqrt[4]{\frac{l_{uh}}{z_{div}}} r_{zdiv}\right)^2 - r_o^2 & \text{if } y \geq 0 \\ x^2 + \left(y + \sqrt[4]{\frac{l_{uh}}{z_{div}}} r_{zdiv}\right)^2 - r_o^2 & \text{if } y < 0 \end{cases} . \quad \textbf{(S24)}$$

A8. Concatenation. Dependent on the z-position of an agent (described below in Supplementary Note B) one of the compartment equations (Eqs S1 and S9 and S12 and S17 and S20 and S22 and S24) is evaluated. Consequently, the compartments are connected in z-direction creating an enclosed volume. Equation Eq S25 defines in which case which equation is evaluated.

$$f(x) = \begin{cases} f_v & \text{if } 0 \leq z < z_{cv} \\ f_{cv} & \text{if } z_{cv} \leq z < z_c \\ f_c & \text{if } z_c \leq z < z_{ub} \\ f_{ub} & \text{if } z_{ub} \leq z < z_{uh} \\ f_{uh} & \text{if } z_{uh} \leq z < z_{utj} \\ f_{utj} & \text{if } z_{utj} \leq z < z_o \\ f_o & \text{if } z_o \leq z \end{cases} \quad \textbf{(S25)}$$

Supplementary Note B: Sperm movement

Basic sperm movement is described by a constraint random walk. In each time-step Δt a new deflection angle $\theta_{s,t}$ and a new speed $v_{s,t}$ are drawn for each individual agent from normal distributions (S4 Table). The deflection angle is drawn from a normal distribution with mean 0° and standard deviation $\theta_{s,t}^{SD\prime}$. Thus, $\theta_{s,t}^{SD\prime}$ depicts how linear an agent is moving. In the absence of thigmotaxis (Supplementary Note B1) $\theta_{s,t}^{SD\prime}$ equals θ_s^{SD}.
Each agent gets its individual θ_s^{SD} from a uniform distribution, within the limits $P_{\theta_s}^{min}$ and $P_{\theta_s}^{max}$, S2 Table and S3 Table. The individual average speed v_s^{avg} of each agent is drawn from a normal distribution with mean $P_{v_s}^{avg}$ and standard deviation $P_{v_s}^{SD}$, S2 Table. In each time-step an agent's orientation $\vec{u}_{s,t}$ is changes by $\theta_{s,t}$, resulting in a new orientation. Afterwards, the position is updated by equation Eq S26.

$$\vec{p}_{s,t+1} = \vec{p}_{s,t} + \vec{u}_{s,t} \cdot v_{s,t} \cdot \Delta t \quad \textbf{(S26)}$$

where, $\vec{p}_{s,t+1}$ is the updated sperm position. If the new position lies within the volume described in Supplementary Note A the new position is accepted, otherwise the former position is kept. A position lies within the described volume if the function value of $f \leq 0$ (Eq S25).

B1. Thigmotaxis. As argued in the main text, sperms can be classified as hydrodynamic pushers, i.e. pushing fluid to the front and to the back, while it is replenished from the sides. Coming close to a wall, the replenishment only takes place from one side and the sperm is pushed and aligned to the wall.
In order to align a sperm's orientation to the closest compartment wall, the closest point on the wall as well as the wall's orientation at this point have to be known. This is realized by defining 14 uniformly distributed points around the sperm position. For each of these points it is investigated if it lies inside or outside of the reproductive tract. If a point lies outside of the reproductive tract, the compartment wall is between that point and the sperm. From all vectors which point to the outside, the average vector is calculated and used as the normal vector of a plane. This plane should be approximately parallel to the

compartment wall. Afterwards the projection of the sperm orientation on the plane is determined. This projection is used to update the orientation by calculating the average of the projection and the orientation.
The same vectors $\vec{cd}$, covering all spatial directions, are used for each sperm in order to investigate, whether the sperm is near a compartment wall.

$$\vec{cd} = \left[\begin{pmatrix}1\\0\\0\end{pmatrix} \begin{pmatrix}0\\1\\0\end{pmatrix} \begin{pmatrix}0\\0\\1\end{pmatrix} \begin{pmatrix}-1\\0\\0\end{pmatrix} \begin{pmatrix}0\\-1\\0\end{pmatrix} \begin{pmatrix}0\\0\\-1\end{pmatrix} \begin{pmatrix}1\\1\\1\end{pmatrix} \begin{pmatrix}1\\1\\-1\end{pmatrix} \begin{pmatrix}1\\-1\\1\end{pmatrix} \begin{pmatrix}-1\\1\\1\end{pmatrix} \begin{pmatrix}1\\-1\\-1\end{pmatrix} \begin{pmatrix}-1\\1\\-1\end{pmatrix} \begin{pmatrix}-1\\-1\\1\end{pmatrix} \begin{pmatrix}-1\\-1\\-1\end{pmatrix} \right]$$

These vectors are normalized and then scaled by (i) their scalar product with the sperm orientation vector and (ii) with half of the length of the sperm. By scaling with the scalar product, vectors which point in a similar direction as the sperm, become longer than those being nearly perpendicular to the sperm orientation. This gives the sperms an ellipsoidal "sensing" zone (S4 Figure, Panel B), which depends on the sperm's length l_s. Additionally, the length of vectors pointing backwards is reduced to a tenth of their original length, preventing that agents get stuck in corners. Consequently sperms sense walls in front of them earlier than walls on their sides/rear. Also, the length of the sperm plays a role, since longer sperms begin to align earlier. Consequently, each sperm has a set of 14 individual scaled vectors for which it should be checked, whether the sum of each vector with the sperm's position would lie outside the reproductive tract. The vectors pointing outside the reproductive tract are now averaged and normalized (S4 Figure, Panel C). The resulting vector is used as the normal vector of a plane, which is approximately parallel to the compartment wall. Next the projection of the sperm's orientation on this plane is calculated (Eq S27).

$$\vec{n_\perp} = \vec{u}_{s,t} - \vec{n}(\vec{n} \cdot \vec{u}_{s,t}) \tag{S27}$$

The new sperm orientation is calculated from the former sperm orientation and the projection by Eq S28, (S4 Figure, Panel D).

$$\vec{u}_{s,t+1} = \frac{\vec{u}_{s,t} + (2^{\frac{\Delta t}{1\,\mathrm{s}}} - 1)\frac{\vec{n_\perp}}{|\vec{n_\perp}|^2}}{2^{\frac{\Delta t}{1\,\mathrm{s}}}} \tag{S28}$$

where $\vec{u}_{s,t}$ is the sperm orientation and $\vec{n}_\perp$ is its projection on the plane. The projection is divided by the square of its length, such that alignment increases the more perpendicular the sperm is to the plane. The term $2^{\frac{\Delta t}{1\,\mathrm{s}}}$ approximates time-step independent alignment.
Before the orientation is updated, an alignment score is calculated by the scalar product of the sperm orientation and the projection on the wall, see Eq S29. This alignment score decreases θ_s^{SD} by Eq S30. As mentioned before without thigmotaxis $\theta_{s,t}^{SD\prime}$ equals θ_s^{SD}, which is the case when $s_{align} = 0$.

$$s_t^{align} = \vec{u}_{s,t} \cdot \vec{n}_\perp \tag{S29}$$

$$\theta_{s,t}^{SD\prime} = \theta_s^{SD} e^{-k_\theta s_t^{align}} \tag{S30}$$

Here, k_θ is a constant defining how strong θ_s^{SD} is reduced due to alignment to the surface.

B2. Fluid Field and positive rheotaxis. For the definition of the flow field of fluid within the female genital tract we make four assumptions:

1. No flow through the UTJ
2. Flow starting below the UTJ, i.e. in the UTJ-compartment with a fixed flow velocity at the lower end of the cervix
3. A continuous fluid volume profile, with a maximal fluid production in the middle of the cervix
4. A Poiseuille like flow, implying that the velocity increases quadratic with the distance to the compartment boundary

The first point requires that the fluid velocity at the transition between oviducts and UTJ equals 0 (that is a z_o). The second point corresponds to the definition of a fluid velocity at the lower end of the cervix (z_c). The third point requires the definition of a profile describing the change in volume flowing through the system dependent on the z position in the system, from this the fluid velocity at each point in the system can be calculated. The fourth point requires an expression of the distance to the compartment wall. Here, the distance is the shortest distance to the compartment wall on a certain height.

Defining the distance to the compartment boundary For the circular compartments, i.e. the compartment without folds, the distance is easy to calculate, see Eq S31.

$$d = \left| \sqrt{x^2 + y^2} - r \right| \tag{S31}$$

For the other compartments the distance was calculated numerically at pre-defined z-positions. The calculation of the distance was performed in two steps. First, finding equidistant points on the compartment boundary, i.e. on the nullcline of the compartment defining function and second, calculating the minimal distance of every point on the xy-plane to every point on the nullcline. Therefore, we had to define two additional parameters, the resolution in z direction z_{sol} and the resolution in x and y direction xy_{sol}. In order to find equidistant points on the nullcline, we used the marching squares algorithm implemented in the skimage.measure.find_contours function.

Maximal fluid velocity For two points in the system, we defined the maximal fluid velocity. First, at the transition from the UTJ to the oviduct compartment and second at the transition from the cranial vagina to the cervix compartment. We assume that there is no fluid flux within the transition from oviducts to UTJ. Further, at the transition from cranial vagina to cervix we define a certain velocity, which can be defined for each simulation individually. In order to calculate the fluid velocity at each point in the system we used the recipe below.

1. Calculate the average velocity at the defined z - positions
2. Calculate the volume flow at each of the two z - position (average velocity times area)
3. Use the two volume flow values to define a volume flow profile (here logistic, which maximal change in the middle of the cervix), which defines a continuous volume flow through the entire system
4. Calculate the average fluid velocity in dependence on z from the continuous volume profile
5. Calculate the maximal fluid velocity from the average fluid velocity

Average velocity at two heights For the UTJ it is straight forward to calculate the average velocity from the maximal velocity by integrating Eq S39 over the radius r_{o_1} and angle ϕ (from 0 to 2π), yielding an average velocity defined by Eq S32:

$$v_{avg} = \frac{v_f^{max}}{2} \pi r_{com}^2 \quad , \tag{S32}$$

where v^{avg} is the average fluid velocity at a certain height in the system. For the transition from cranial vagina to cervix the average velocity was calculated numerically, by averaging over the array given by Eq S40. Next, the average velocities were multiplied by the cross-sectional area at the given heights (z_o and z_c), resulting in the volume flow through this cross-section. These two points were afterwards used to define the continuous volume flow profile (see S6 Figure). Thereafter, the area in dependence of z was calculated. For the vagina, cranial vagina, cervix and uterine body compartment the area was given by $A = \pi r_{com}^2$. In the case of the uterine horns one has to distinguish between two cases: (1) the area below the height at which two tubes emerge and (2) the area above that point. The area above is simply given by $A = 2\pi r_{uh}^2$, as the uterine horns consist of two circular tubes. For the part of the uterine horns below the division point the cross-section is equal to two overlapping circles and calculated by the following equations:

$$s = 2\sqrt{r^2 - y_m^2} \tag{S33}$$

$$\alpha = 2 \arcsin \frac{s}{2r} \tag{S34}$$

$$A_B = \pi r^2 \frac{\alpha}{360} \tag{S35}$$

$$A_d = \frac{y_m s}{2} \tag{S36}$$

$$A_i = A_B - A_d \tag{S37}$$

$$A_{ub} = 2\pi r^2 - 2A_i \tag{S38}$$

where s is the distance between the two contact points of the circle, α is the angle spanned by those points and one of the midpoints and A_B is the area of the segment of the circle defined by the midpoint, see S5 Figure. A_i and A_d are the green and orange areas in S5 Figure, respectively.

Dividing the volume flow through the cross-sectional area results in the average fluid velocity in dependence on z. The maximal fluid velocity follows from Eq S32 or by dividing the average velocity by the numerical average of the Poiseuille profile at this z position, which corresponds to the average velocity resulting from a maximal velocity of unity. S6 Figure shows the defined volume flow, the cross-sectional area, the average velocity and finally the maximal velocity in dependence on the z position.

Poiseuille Profile Having the distance to the compartment wall defined we can calculate the fluid velocity at each point by Eqs S39 and S40, obtaining an analytically or numerically calculated distance respectively.

$$v_f = v_f^{max}\left(1 - \frac{r^2}{r_{com}^2}\right) \quad \textbf{(S39)}$$

$$v_f = v_f^{max}\left(1 - \frac{(d_{max} - d(x,y))^2}{d_{max}^2}\right) \quad \textbf{(S40)}$$

Here, r_{com} stands for the radius of the system at a certain height, as defined by the different compartment functions. d_{max} is the maximal distance to the compartment wall and $d(x,y)$ the distance to the wall at the point (x,y). The only unknown entity is the maximal fluid velocity v_f^{max}. This maximal fluid velocity depends on the z - position and the next paragraph summarizes the underlying assumptions.
Within the box compartment used to simulate sperms in a specimen chamber the flow profile was calculated with the solution of the Poiseuille equation for the rectangular case [1] , Eq S41. The profile is visualized in Fig 2I.

$$v_f(y,z) = v_f^{max} \cdot \sum_{i=0}^{\infty} \frac{(-1)^n}{(2n+1)^3} \cdot \left(1 - \frac{cosh\left(\frac{(2n+1)\pi y}{a}\right)}{cosh\left(\frac{(2n+1)\pi b}{2a}\right)}\right) \cdot cos\left((2n+1)\pi\frac{z}{a}\right) \quad \textbf{(S41)}$$

We used $n = 1$ as additional terms of the series do not change the velocity field significantly. The direction of the flow is defined to be in x-direction, thus the flow velocity depends only on y and z.
Using agent velocities from Hyakutake et al. [2], who measured bovine sperm velocities in cervical mucus like medium, it was indirectly assumed that the mucus is a Non-Newtonian fluid. The assumed Poiseuille Profile is valid only for Newtonian fluids. For Non-Newtonian fluids the decrease in fluid velocity towards the boundaries becomes steeper [3] , keeping the general appearance of the profile. Therefore the positive effect of positive rheotaxis might even be underestimated.

Fluid flow direction The fluid direction is defined compartment-wise. In the vagina and the cervix, which do not change the radius with height, the fluid is directed down the z-axis. The other compartments are of conical shape and, therefore, the fluid is directed away from or towards the center of the cone, see sketch S7 Figure. For the cranial vagina and the uterine body, the position of the center depends on the primary folds. The center can be defined using the second intercept theorem, Eqs S42-S46.

$$\beta = \frac{r_u}{r_l} \quad \textbf{(S42)}$$

$$\beta = \begin{cases} \frac{z_{center}}{z_{center}+l_{com}} & \text{if } \beta < 1 \\ \frac{z_{center}+l_{com}}{z_{center}} & \text{if } \beta > 1 \end{cases} \quad \textbf{(S43)}$$

$$z_{center} = \begin{cases} \frac{\beta l_{com}}{1-\beta} & \text{if } \beta < 1 \\ \frac{l_{com}}{\beta - 1} & \text{if } \beta > 1 \end{cases} \quad \textbf{(S44)}$$

$$\vec{c} = \begin{cases} (x_m, y_m, z_{offset} + z_{center})^T & \text{if } \beta < 1 \\ (x_m, y_m, z_{offset} - z_{center})^T & \text{if } \beta > 1 \end{cases} \quad \textbf{(S45)}$$

$$\vec{u_f} = \begin{cases} \vec{p}_{s,t} - \vec{c} & \text{if } \beta < 1 \\ \vec{c} - \vec{p}_{s,t} & \text{if } \beta > 1 \end{cases} \quad \textbf{(S46)}$$

In most cases x_m and y_m are 0. Only in the UTJ compartment y_m is calculated as in Eq S22. $\vec{p}_{s,t}$ is the position vector of a sperm and $\vec{u}_f$ the resulting fluid direction vector, which is normalized.

Positive Rheotaxis As discussed in the main text, sperms align into the fluid flow. Here, we make the following assumptions. First, faster sperms align quicker and second, faster fluid flow leads to faster sperm alignment. The fluid direction vector $\vec{u}_f$ is scaled by Eq S47 (sf for scaling factor). The sperm orientation $\vec{u}_{s,t}$ is updated by averaging $\vec{u}_f$ and $\vec{u}_{s,t}$ in a time-step independent manner, see Eq S48.

$$sf = \frac{v_s}{P_{v_s}^{avg}} \frac{v_f}{v_f^0} \left(\frac{1}{1+e^{-f_k(v_f - v_f^0)}}\right) \cdot \|\vec{u_f} \times \vec{u}_{s,t}\| \quad \textbf{(S47)}$$

$$\vec{u}_{s,t+1} = \frac{\vec{u}_{s,t} - (2^{\Delta t} - 1)\vec{u_f} sf}{2^{\Delta t}} \quad \textbf{(S48)}$$

Here, $P_{v_s}^{avg}$ is the average sperm velocity, v_f^0 is the fluid velocity at which alignment occurs and f_k is steepness of the alignment response. The logistic term was chosen as it was reported that sperms only align in sufficiently fast fluid flows.

Supplementary Note C: Immune system

Each agent possesses an individual life time τ_{ls}, drawn from a normal distribution with mean $P_{\tau_{ls}}^{avg}$ and standard deviation $P_{\tau_{ls}}^{SD}$ (S2 Table and S3 Table). The immune system is described as a decrease in the life time. The immune system activity is described by a Hill function with a half maximal activity after 4 hours and a Hill coefficient of 2 (S8 Figure). Given the maximal immune system strength and a default life time of 86400 seconds (1 day) this would be reduced to 30 minutes. Mullins and Saacke proposed that sperms within secondary folds (microgrooves) could be protected from the immune system [4]. Therefore agents located in microgrooves do not experience life time reduction by the immune system (S8 Figure).

Supplementary Note D: Sperm persistence

Although elaborated sperm tracking techniques exist [5, 6] and a descriptive set of movement parameters is defined within the Computer Assisted Sperm Analysis (CASA) framework [7], knowledge is sparse on the angular deflection of sperm per time. Within the CASA framework, straightness (STR) is calculated by the vector length of displacement divided by the contour length of a sperm trajectory. Tung et al.[8] measured STR values of 0.87 ± 0.02 for bovine sperm tracked for $2.81\,\mathrm{s}$. Using this value we estimated the deflection angles for sperm movement, by simulating agent movement in a box of height $120\,\mathrm{\mu m}$ and calculating the persistence [8, 9]. The agents were positioned in the middle of the chamber, restricting movement to z-direction. Calculation of the straightness showed that (i) it is timestep independent, which is ensured by the Euler-Maruyama method [10], and (ii) that it perfectly agrees with the measurements from Tung et al. [8](S9 Figure). Sperm agents were simulated in the box, with different time-step lengths Δt ($0.01\,\mathrm{s}$, $0.1\,\mathrm{s}$, $0.5\,\mathrm{s}$ and $1\,\mathrm{s}$). We first simulated 50 agents for $2.81\,\mathrm{s}$ in order to compare agent persistence with sperm persistence measured by Tung et al.[8](0.87 ± 0.02). We adjusted the limits of the uniform distribution from which θ_s^{SD} is drawn, such that the persistence of agents were similar to the measured data. Second, we simulated for $3\,\mathrm{s}$ with different time steps in order to validate that persistence is time-step independent. Comparison of the simulations with different time-steps showed that the persistence is time-step independent, S9 Figure.

Supplementary Note E: Thigmotaxis aids transition through cervix and UTJ

Analysis revealed that wall alignment is especially important while passing the cervix and the UTJ, S10 Figure. To investigate if the positive effect of thigmotaxis is a direct consequence of wall alignment or if if thigmotaxis helps the agents to evade the immune system by hiding in microgrooves we performed simulations without immune system and a simulation time of 48 h, S11 Figure. As no agent reaches the oviducts without thigmotaxis, but $\approx 0.25\,\%$ do with thigmotaxis, this motility behaviour alone is responsible for more successful agents.

Supplementary Note F: Computational Execution

The model was implemented in *Python*. In order to visualize agent movement a *vtk* (Visualization Toolkit [11]) export was implemented, such that the data could be visualized later in Paraview [12]. The simulation was parallelized by using a bash script, which started the simulation on different cores.

Supplementary References

1. Franck Delplace. Laminar flow of Newtonian liquids in ducts of rectangular cross-section a model for both physics and mathematics. *Open Access Journal of Mathematical and Theoretical Physics*, 1(5):198–201, sep 2018. doi: 10.15406/oajmtp.2018.01.00034.
2. Toru Hyakutake, Kotaro Mori, and Koichi Sato. Effects of surrounding fluid on motility of hyperactivated bovine sperm. *Journal of Biomechanics*, 71:183–189, apr 2018. ISSN 18732380. doi: 10.1016/j.jbiomech.2018.02.009.
3. Byung Chan Eu. Generalization of the Hagen–Poiseuille velocity profile to non-Newtonian fluids and measurement of their viscosity. *American Journal of Physics*, 58(1):83–84, jan 1990. ISSN 0002-9505. doi: 10.1119/1.16328.
4. K. June Mullins and R. G. Saacke. Study of the functional anatomy of bovine cervical mucosa with special reference to mucus secretion and sperm transport. *The Anatomical Record*, 225(2): 106–117, oct 1989. ISSN 10970185. doi: 10.1002/ar.1092250205.
5. Ting-Wei Su, Liang Xue, and Aydogan Ozcan. High-throughput lensfree 3D tracking of human sperms reveals rare statistics of helical trajectories. *Proceedings of the National Academy of Sciences of the United States of America*, 109(40):16018–22, oct 2012. ISSN 1091-6490. doi: 10.1073/pnas.1212506109.
6. Leonardo F. Urbano, Puneet Masson, Matthew VerMilyea, and Moshe Kam. Automatic Tracking and Motility Analysis of Human Sperm in Time-Lapse Images. *IEEE Transactions on Medical Imaging*, 36(3):792–801, mar 2017. ISSN 0278-0062. doi: 10.1109/TMI.2016.2630720.
7. Rupert P. Amann and Dagmar Waberski. Computer-assisted sperm analysis (CASA): Capabilities and potential developments. *Theriogenology*, 81(1):5–17, 2014. ISSN 0093691X. doi: 10.1016/j.theriogenology.2013.09.004.
8. Chih-kuan Tung, Lian Hu, Alyssa G. Fiore, Florencia Ardon, Dillon G. Hickman, Robert O. Gilbert, Susan S. Suarez, and Mingming Wu. Microgrooves and fluid flows provide preferential passageways for sperm over pathogen <i>Tritrichomonas foetus</i>. *Proceedings of the National Academy of Sciences*, 112(17):5431–5436, apr 2015. ISSN 0027-8424. doi: 10.1073/pnas.1500541112.
9. Laura M King, Denise R Holsberger, and Ann M Donoghue. Correlation of CASA Velocity and Linearity Parameters With Sperm Mobility Phenotype in Turkeys. *Journal of Andrology J Androl*, 2121 (1):65–71, jan 2000. ISSN 0196-3635. doi: 10.1002/j.1939-4640.2000.tb03277.x.
10. P E Kloeden and E Platen. *Numerical Solution of Stochastic Differential Equations*, volume 23. Springer Berlin Heidelberg, Berlin, Heidelberg, 1995. ISBN 978-3-642-08107-1. doi: 10.1007/978-3-662-12616-5.
11. W Schroeder, K Martin, and B Lorensen. *The visualization toolkit: an object oriented approach to 3D graphics. New York: Kitware.* Kitware, 2006. ISBN 9781930934191.
12. Utkarsh Ayachit. *The ParaView Guide: A Parallel Visualization Application.* Kitware, Inc., USA, 2015. ISBN 1930934300 9781930934306. doi: citeulike-article-id:4375994.

13. Busch and Waberski. *Künstliche Besamung bei Haus- und Nutztieren*. Schattauer, 2007. ISBN 3-334-00379-5.
14. J M Cummins and P F Woodall. On mammalian sperm dimensions. *Journal of reproduction and fertility*, 75(1):153–175, sep 1985. ISSN 1470-1626. doi: 10.1530/jrf.0.0750153.
15. Pauli Virtanen, Ralf Gommers, Travis E Oliphant, Matt Haberland, Tyler Reddy, David Cournapeau, Evgeni Burovski, Pearu Peterson, Warren Weckesser, Jonathan Bright, Stéfan J van der Walt, Matthew Brett, Joshua Wilson, K Jarrod Millman, Nikolay Mayorov, Andrew R J Nelson, Eric Jones, Robert Kern, Eric Larson, C J Carey, İlhan Polat, Yu Feng, Eric W Moore, Jake VanderPlas, Denis Laxalde, Josef Perktold, Robert Cimrman, Ian Henriksen, E A Quintero, Charles R Harris, Anne M Archibald, Antônio H Ribeiro, Fabian Pedregosa, Paul van Mulbregt, Aditya Vijaykumar, Alessandro Pietro Bardelli, Alex Rothberg, Andreas Hilboll, Andreas Kloeckner, Anthony Scopatz, Antony Lee, Ariel Rokem, C Nathan Woods, Chad Fulton, Charles Masson, Christian Häggström, Clark Fitzgerald, David A Nicholson, David R Hagen, Dmitrii V Pasechnik, Emanuele Olivetti, Eric Martin, Eric Wieser, Fabrice Silva, Felix Lenders, Florian Wilhelm, G Young, Gavin A Price, Gert-Ludwig Ingold, Gregory E Allen, Gregory R Lee, Hervé Audren, Irvin Probst, Jörg P Dietrich, Jacob Silterra, James T Webber, Janko Slavič, Joel Nothman, Johannes Buchner, Johannes Kulick, Johannes L Schönberger, José Vinícius de Miranda Cardoso, Joscha Reimer, Joseph Harrington, Juan Luis Cano Rodríguez, Juan Nunez-Iglesias, Justin Kuczynski, Kevin Tritz, Martin Thoma, Matthew Newville, Matthias Kümmerer, Maximilian Bolingbroke, Michael Tartre, Mikhail Pak, Nathaniel J Smith, Nikolai Nowaczyk, Nikolay Shebanov, Oleksandr Pavlyk, Per A Brodtkorb, Perry Lee, Robert T McGibbon, Roman Feldbauer, Sam Lewis, Sam Tygier, Scott Sievert, Sebastiano Vigna, Stefan Peterson, Surhud More, Tadeusz Pudlik, Takuya Oshima, Thomas J Pingel, Thomas P Robitaille, Thomas Spura, Thouis R Jones, Tim Cera, Tim Leslie, Tiziano Zito, Tom Krauss, Utkarsh Upadhyay, Yaroslav O Halchenko, Yoshiki Vázquez-Baeza, and SciPy 1.0 Contributors. SciPy 1.0: fundamental algorithms for scientific computing in Python. *Nature Methods*, 17(3):261–272, 2020. ISSN 1548-7105. doi: 10.1038/s41592-019-0686-2.
