# Supplementary material for: Sperm migration in the genital tract—In silico experiments identify key factors for reproductive success
Source: PLoS Comput Biol. 2021 Jul 15;17(7):e1009109. doi: 10.1371/journal.pcbi.1009109 (PMC8282070; doi:10.1371/journal.pcbi.1009109)
Supplement: S1 Table — Dimensional parameters were taken from Busch et al. [48]. Educated guesses for the number of microgrooves were taken with the help of microscopic images and sketches from Mullins and Saacke [21]. (PDF) [file pcbi.1009109.s002.pdf]

S1 Table. Parameters used in implicit functions for description of the bovine female genital tract. Dimensional parameters were taken from Busch et al. [48]. Educated guesses for the number of microgrooves were taken with the help of microscopic images and sketches from Mullins and Saacke [21].

Parameter	Value	Description	Source
l_v	25 cm	Length of the vagina	Busch et al.[48]
r_v	2.5 cm	Radius of the vagina	
l_{cv}	5 cm	Length of the cranial vagina	
r_c	1 cm	Radius of the cervix	
l_c	8 cm	Length of the cervix	Busch et al.[48]
l_{ub}	3 cm	Length of the uterine body	Busch et al.[48]
r_{uh_1}	1.5 cm	Radius at lower end of uterine horns	
r_{uh_2}	0.5 cm	Radius at the upper end of uterine horns	
l_{uh}	40 cm	Length of the uterine horns	Busch et al.[48]
l_{utj}	5 cm	Length of the UTJ	
l_o	25 cm	Length of oviducts	Busch et al.[48]
r_{o_1}	0.05 cm	Radius at lower end of oviducts	
r_{o_2}	0.1 cm	Radius upper end of oviducts	
z_{cv}	25 cm	z baseline of cranial vagina	calculated
z_c	30 cm	z baseline of cervix	calculated
z_{ub}	38 cm	z baseline of uterine body	calculated
z_{uh}	41 cm	z baseline of uterine horns	calculated
z_{utj}	81 cm	z baseline of UTJ	calculated
z_o	86 cm	z baseline of oviducts	calculated
d_{center}	-	Distance to z-axis	calculated (Eq S3)
d_r	-	Distance to compartment radius	calculated (Eq S10)
A_{pf}	-	Amplitude of primary folds	calculated (Eq S6
A_{pf}^{max}	-	Maximal depth of primary folds.	calculated (Eq S5)
A_{sf}	-	Amplitude of secondary folds	calculated (Eq S7)
A_{sf}^{max}	0.3	Maximal depth of secondary folds.	educated guess([21])
k_A^{sf}	0.8	-	educated guess([21])
k_A^{pf}	0.5	Fraction of primary folds where secondary folds occur	educated guess([21])
ω_{pf}	8	Number of primary folds	educated guess([21])
ω_{sf}	32	Number of secondary folds	educated guess([21])
z_{div}	4	Height at which uterine horns divide	educated guess([21])
r_{zdiv}	-	Radius at which uterine horns divide	calculated (Eq S19)
