# Supplementary material for: Sperm migration in the genital tract—In silico experiments identify key factors for reproductive success
Source: PLoS Comput Biol. 2021 Jul 15;17(7):e1009109. doi: 10.1371/journal.pcbi.1009109 (PMC8282070; doi:10.1371/journal.pcbi.1009109)
Supplement: S2 Table — Parameters were taken from experimental data [19, 49]. (PDF) [file pcbi.1009109.s003.pdf]

S2 Table. Sperm population parameters. Parameters were taken from experimental data [19, 49].

Parameter	Value	Description	Source
$P_{v_s}^{avg}$	$60\,\mu m/s$	Population mean of sperm speed	Hyakutake et al.[19]
$P_{v_s}^{SD}$	$10\,\mu m/s$	Population standard deviation of sperm speed	Hyakutake et al.[19]
$P_{\tau_{ls}}^{avg}$	$86\,400\,s$	Population mean of sperm lifetime	educated guess
$P_{\tau_{ls}}^{SD}$	$21\,600\,s$	Population standard deviation of sperm lifetime	educated guess
$P_{\theta_s}^{min}$	$1°$	Minimal standard deviation of angle distribution	Comparison with Tung et al. [20] (supplementary note 4)
$P_{\theta_s}^{max}$	$119°$	Maximal standard deviation of angle distribution	Comparison with Tung et al. [20] (supplementary note 4)
$P_{l_s}^{avg}$	$65\,\mu m$	Average sperm length	$50\,\mu m$ to $80\,\mu m$ Cummins & Woodall[49]
$P_{l_s}^{SD}$	$5\,\mu m$	Standard deviation of sperm length	educated guess
