# Supplementary material for: Sperm migration in the genital tract—In silico experiments identify key factors for reproductive success
Source: PLoS Comput Biol. 2021 Jul 15;17(7):e1009109. doi: 10.1371/journal.pcbi.1009109 (PMC8282070; doi:10.1371/journal.pcbi.1009109)
Supplement: S3 Table — (PDF) [file pcbi.1009109.s004.pdf]

S3 Table. Individual sperm agent parameters.

Parameter	Distribution		Description
v_s^{avg}	$\mathcal{N}(\mu, \sigma^2)$	$\mu = P_{v_s}^{avg}, \sigma = P_{v_s}^{avg}$	Average speed of individual agent
v_s^{SD}	-	$v_s^{SD} = v_s^{avg} 10^{-1}$	Standard deviation of speed of individual agent
θ_s^{avg}	-	0	Average angle of individual agent
θ_s^{SD}	$\mathcal{U}(a,b)$	$a = P_{\theta_s}^{min}, b = P_{\theta_s}^{max}$	Standard deviation of angle of individual agent
τ_{ls}	$\mathcal{N}(\mu, \sigma^2)$	$\mu = P_{\tau_{ls}}^{avg}, \sigma = P_{\tau_{ls}}^{SD}$	Lifetime of individual agent
l_s	$\mathcal{N}(\mu, \sigma^2)$	$\mu = P_{l_s}^{avg}, \sigma = P_{l_s}^{SD}$	Length of individual agent
