# Supplementary material for: Sperm migration in the genital tract—In silico experiments identify key factors for reproductive success
Source: PLoS Comput Biol. 2021 Jul 15;17(7):e1009109. doi: 10.1371/journal.pcbi.1009109 (PMC8282070; doi:10.1371/journal.pcbi.1009109)
Supplement: S4 Table — (PDF) [file pcbi.1009109.s005.pdf]

S4 Table. Temporary sperm agent and other parameters.

Parameter	Origin		Description
$\theta_{s,t}^{SD\prime}$	Eq S30	-	Diminished standard deviation of individual deflection angle distribution
$\theta_{s,t}$	$\mathcal{N}(\mu, \sigma^2)$	$\mu = 0$, $\sigma = \theta_{s,t}^{SD\prime}$	Deflection angle
$v_{s,t}$	$\mathcal{N}(\mu, \sigma^2)$	$\mu = v_s^{avg}$, $\sigma = v_s^{SD}$	Temporary sperm agent speed
s_t^{align}	Eq S29	-	Score for the alignment with a compartment wall
k_θ	0.5	-	Strength of thigmotaxis
v_f^0	$20\,\mu\mathrm{m/s}$	-	Sperm alignment velocity
f_k	0.5	-	Steepness alignment response
