# Supplementary figures and images for: Sperm migration in the genital tract—In silico experiments identify key factors for reproductive success
Source: PLoS Comput Biol. 2021 Jul 15;17(7):e1009109. doi: 10.1371/journal.pcbi.1009109 (PMC8282070; doi:10.1371/journal.pcbi.1009109)

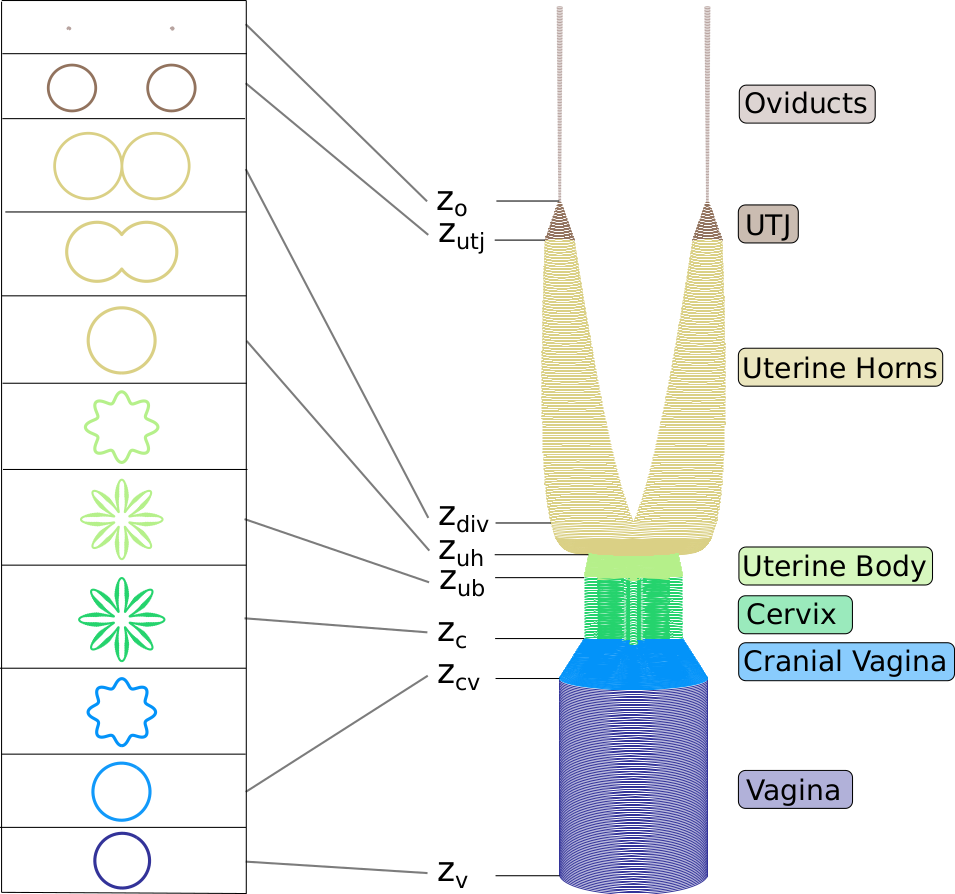

Supplement: S1 Fig — Individual compartments (listed on the right) were connected in z-direction. Labelled z-positions indicate compartment transitions and the z-position at which uterine horns divide (Supplementary Note A in S1 Text). Cross-sections at these and intermediate positions are shown on the left. (TIF) [file pcbi.1009109.s006.tif]

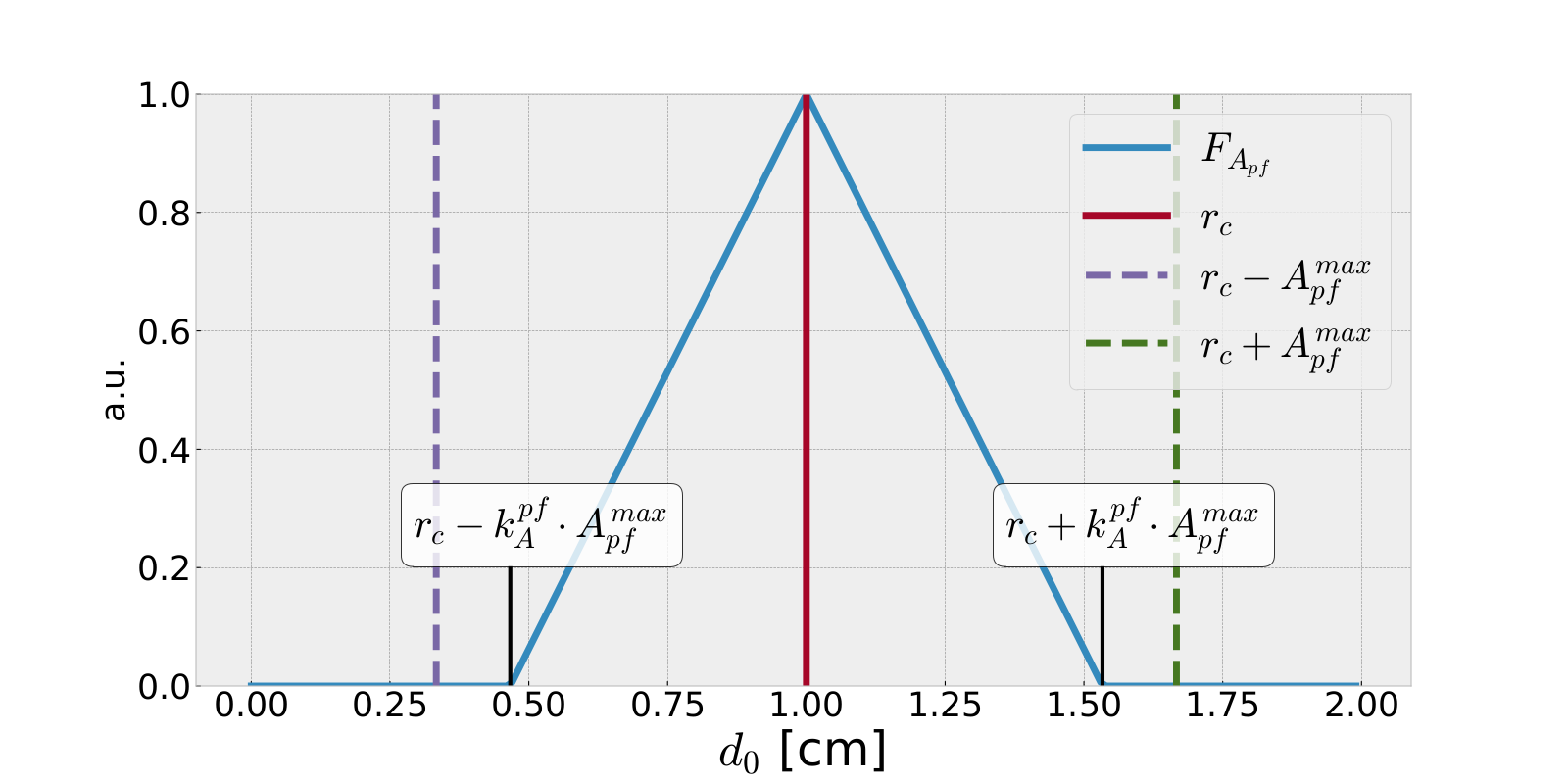

Supplement: S2 Fig — Dashed purple and green line indicate the beginning and end of a primary fold respectively. Red line indicates the center of the primary fold, while the blue line indicates the relative scaling of the secondary fold depth. (TIF) [file pcbi.1009109.s007.tif]

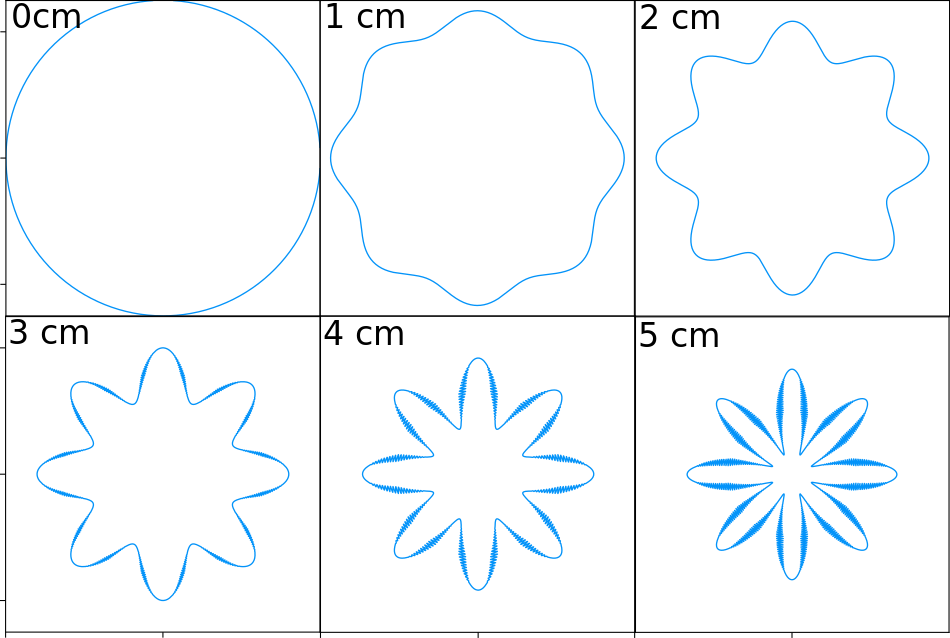

Supplement: S3 Fig — At 0 cm the cranial vagina equals the shape of the vagina, thus the cross-section is a simple circle. With increasing height first the primary (1 cm and 2 cm) and later also the secondary (3 cm, 4 cm and 5 cm) folds develop. Notice that the secondary folds only occur within the upper half of the cranial vagina (restricted in the condition of Eq S7 in S1 Text by kAsf) and only in the center of the primary folds (restricted by FApf). At 5 cm the cross-section equals the cross-section of the cervix. (TIF) [file pcbi.1009109.s008.tif]

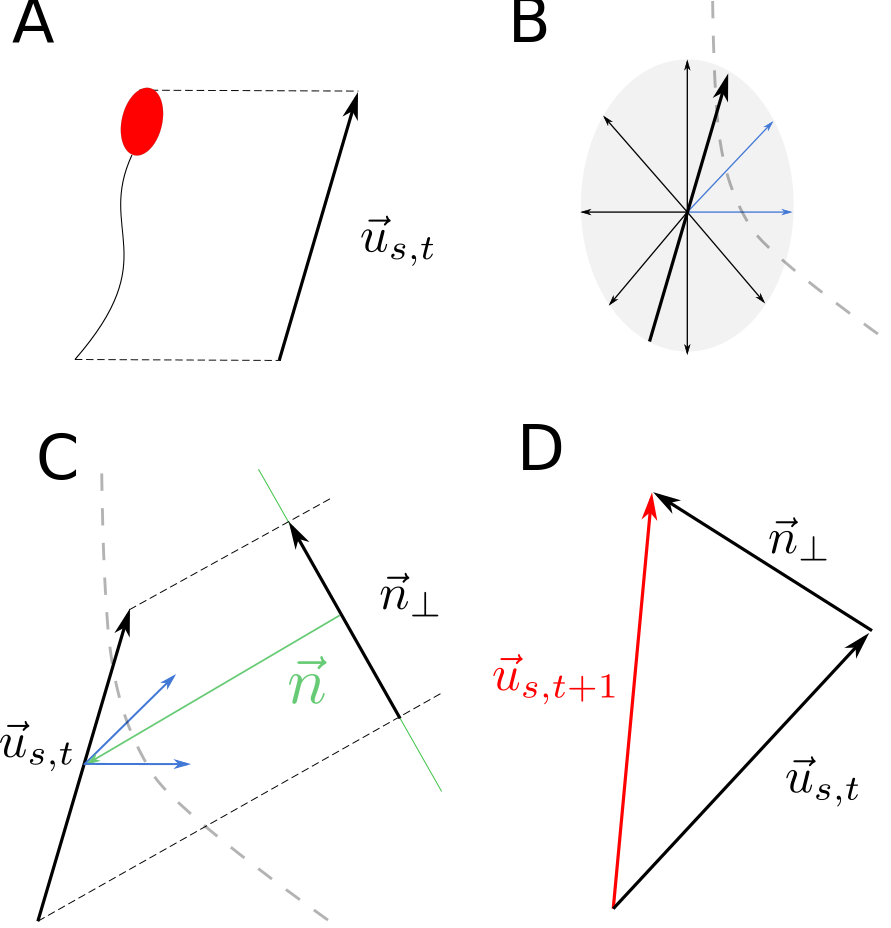

Supplement: S4 Fig — A Sperm orientation is defined by its orientation vector. B The solid black vector indicates sperm orientation. Smaller black and blue arrows indicate the scaled vectors, for which it is checked if they lie inside or outside the reproductive tract. The scaling provokes an ellipsoidal shape, indicated by the light grey shaded ellipse. The dashed grey line mimics a compartment wall. The two blue colored arrows, point outside the compartment. C The weighted average of the vectors pointing outside (shown in blue) of the compartment defines the normal vector of a plane. Sperm orientation as solid black arrow with label u→s,t. Green arrow indicates resulting normal vector n→. For representational reason it was inverted and enlarged. This normal vector described a plane, shown in dark green. n→⊥ depicts the projection from u→s,t onto the plane defined by n→. D The new sperm orientation u→s,t+1 vector is shown in red. It results from the sum of the former direction vector and the projection onto the plane. Subsequent the new orientation vector is normalized. (TIF) [file pcbi.1009109.s009.tif]

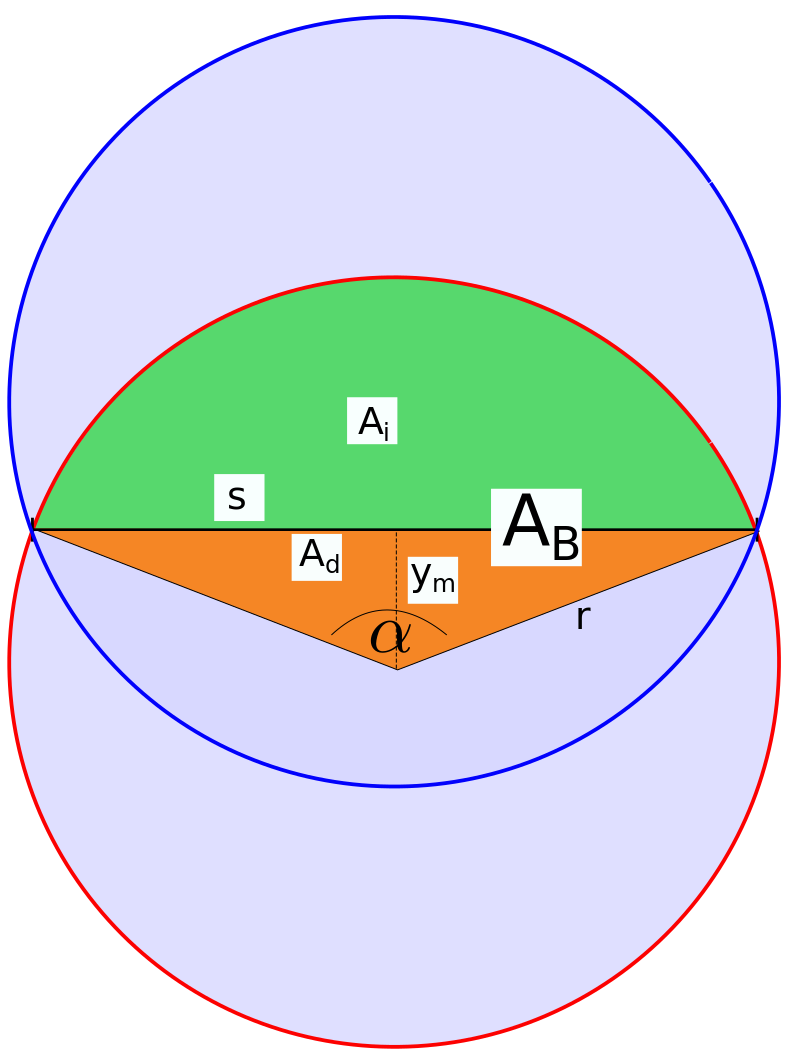

Supplement: S5 Fig — s is the distance between the overlapping points. Ai is the green and Ad the orange area, while AB is the area covered by the radiant α. ym is the distance of one midpoint to the line s. Values calculated by Eqs S33-S38 in Supplementary Note B in S1 Text. (TIF) [file pcbi.1009109.s010.tif]

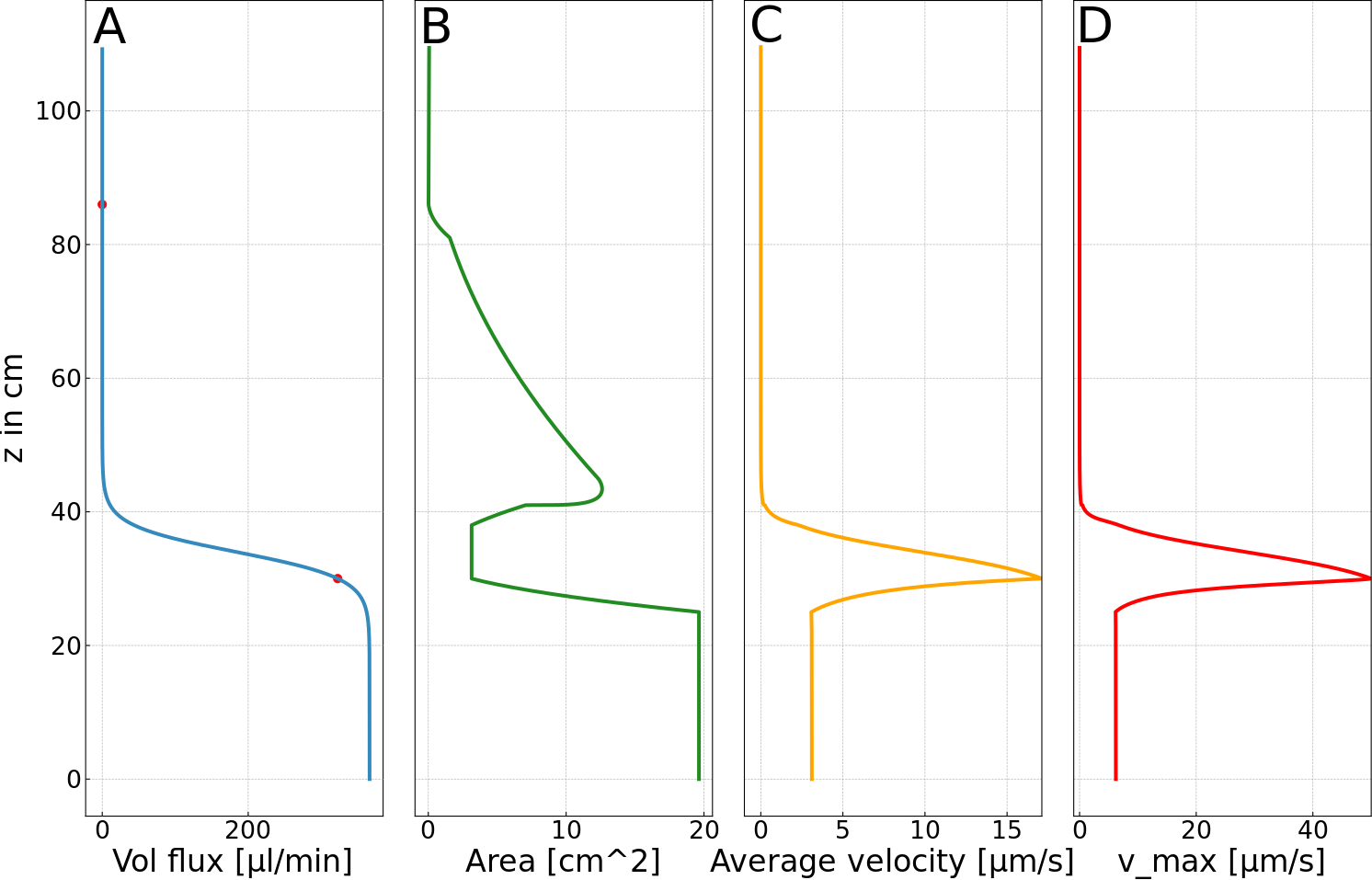

Supplement: S6 Fig — A Volume flow. Red dots indicate heights at which the maximal fluid velocity was set. Blue line corresponds to the continuous volume gain throughout the system. The rate of change is maximal in the middle of the cervix compartment (z = 34 cm); B Cross-sectional area as function of z. C Average fluid velocity calculated from continuous volume flux (A) and cross-sectional are (B). D Maximal fluid velocity as function of z, calculated from the average fluid velocity (C). (TIF) [file pcbi.1009109.s011.tif]

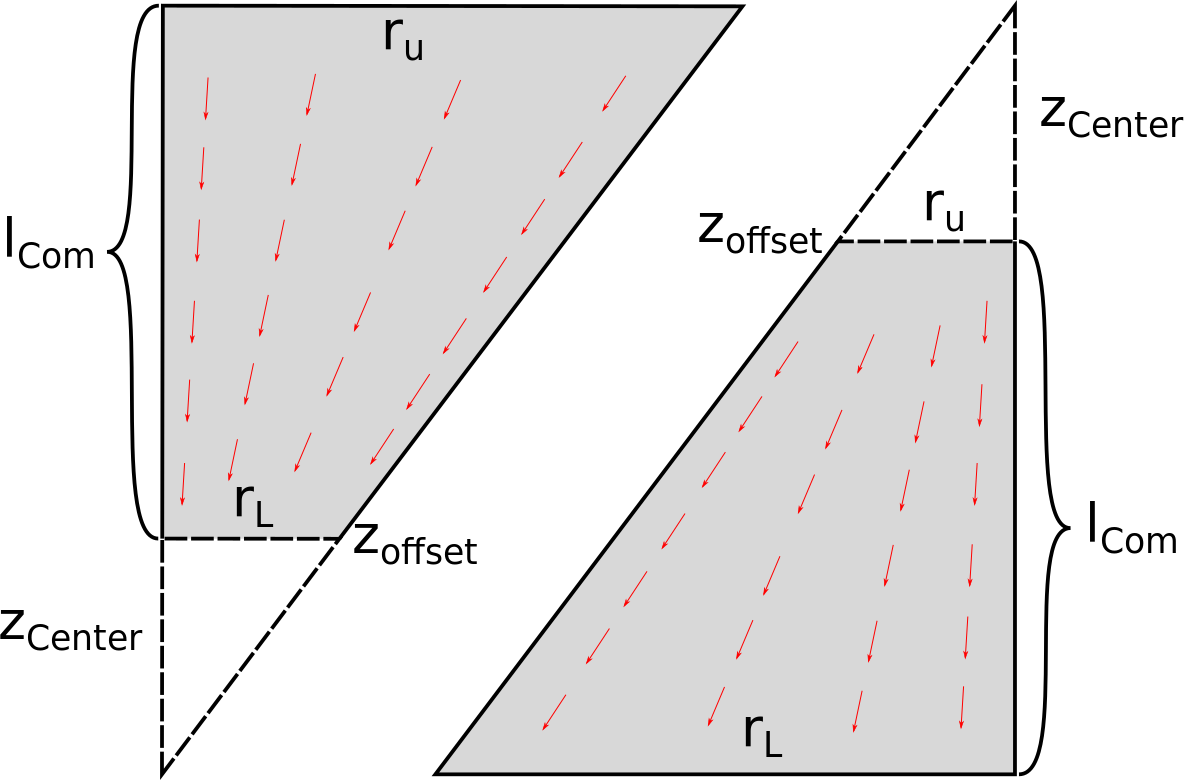

Supplement: S7 Fig — The compartment is depicted by the grey area. rl and ru are the lower and upper compartment radii and lcom the compartment length. zcenter is the distance from the cone center to the compartment boundary zoffset. One has to distinguish between the cases that the upper radius is larger than the lower radius (β > 1) and vice versa (β < 1). In the first case, the fluid flow is directed towards the cone center and in the second case away from the center as depicted by the red arrows. (TIF) [file pcbi.1009109.s012.tif]

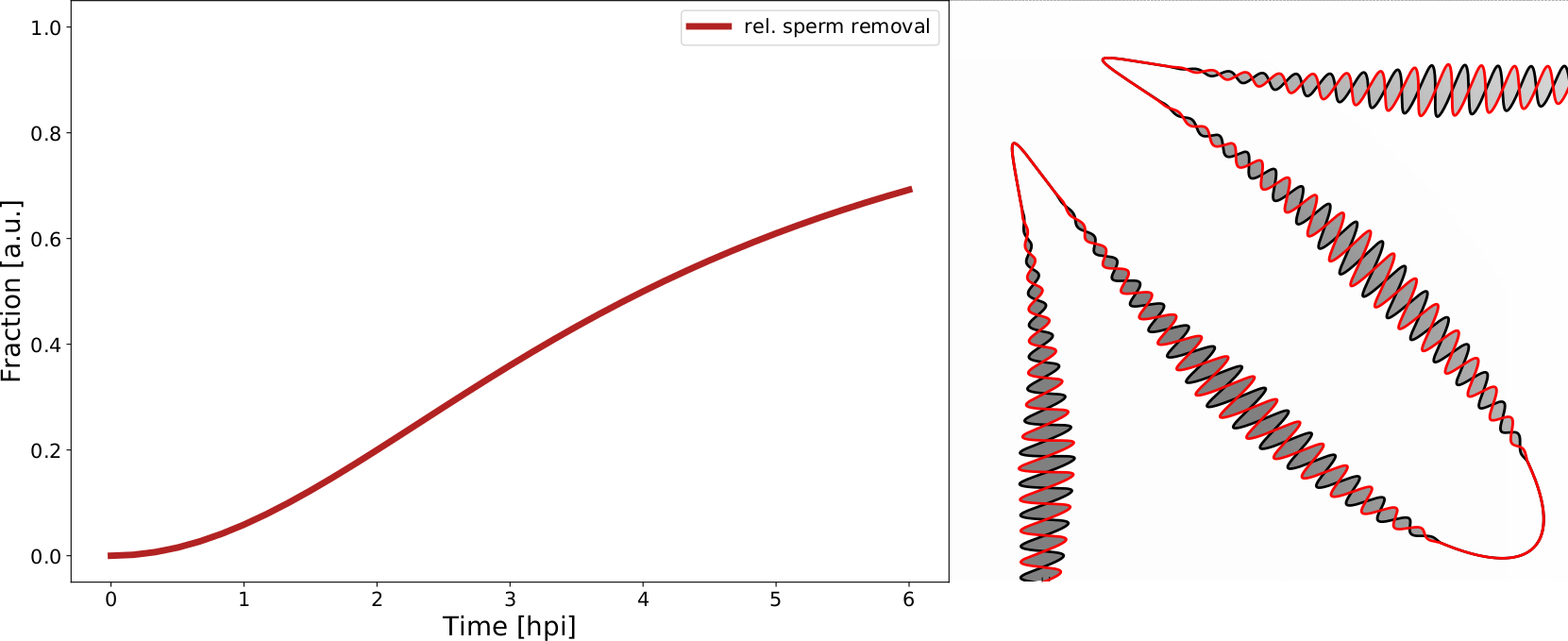

Supplement: S8 Fig — Left: Hill function describing the relative activity of the immune system. Right: Agents within microgrooves are protected from the immune system. An agent is defined to be in a microgroove when it is positioned within the modeled genital tract and outside the female genital tract with inverted secondary folds (Asfmax=−0.3). The figure shows an excerpt of the cervical cross-section of the female genital tract with original (black) and inverted (red) secondary folds. Shaded areas depict the cross-section of microgrooves. (TIF) [file pcbi.1009109.s013.tif]

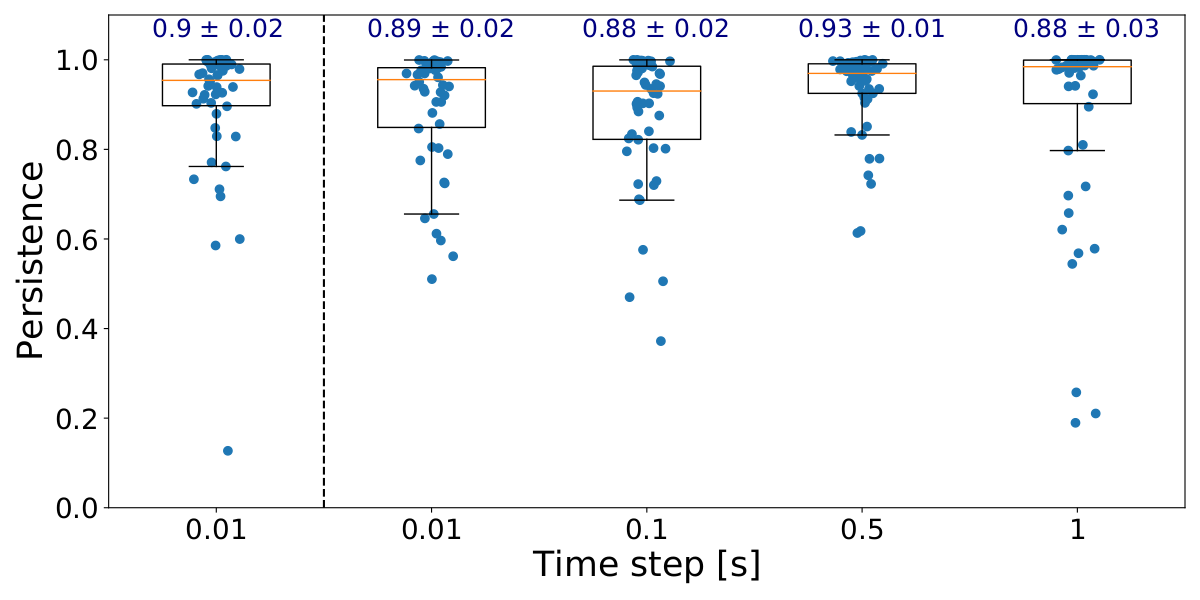

Supplement: S9 Fig — Persistence of simulated agents in a box of 120 μm height. Text above the boxplots gives Mean ± SEM. Orange line indicates median persistence. The persistence shown on the left hand side of the figure originates from a simulation for 2.81 s. For the other persistence the time step was altered and the simulation time was set to 3 s, in order to make persistence comparable between the simulations. Persistence of simulated sperms is in agreement with the persistence reported by Tung et al. [20] (0.87 ± 0.02). (TIF) [file pcbi.1009109.s014.tif]

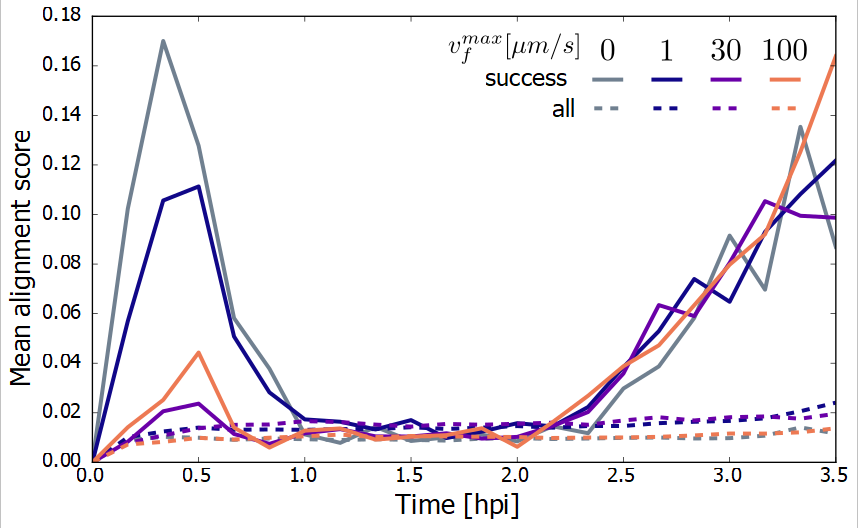

Supplement: S10 Fig — Mean of alignment score stalign over time. Successful sperms are compared to all sperms. Especially for simulations with only thigmotaxis (vfmax=0) alignment to the wall within the first 30 minutes (while passing the cervix) increases the possibility to be successful. Independent of vfmax alignment aids the transit through the UTJ (increased mean alignment after 2 hpi.). (TIF) [file pcbi.1009109.s015.tif]

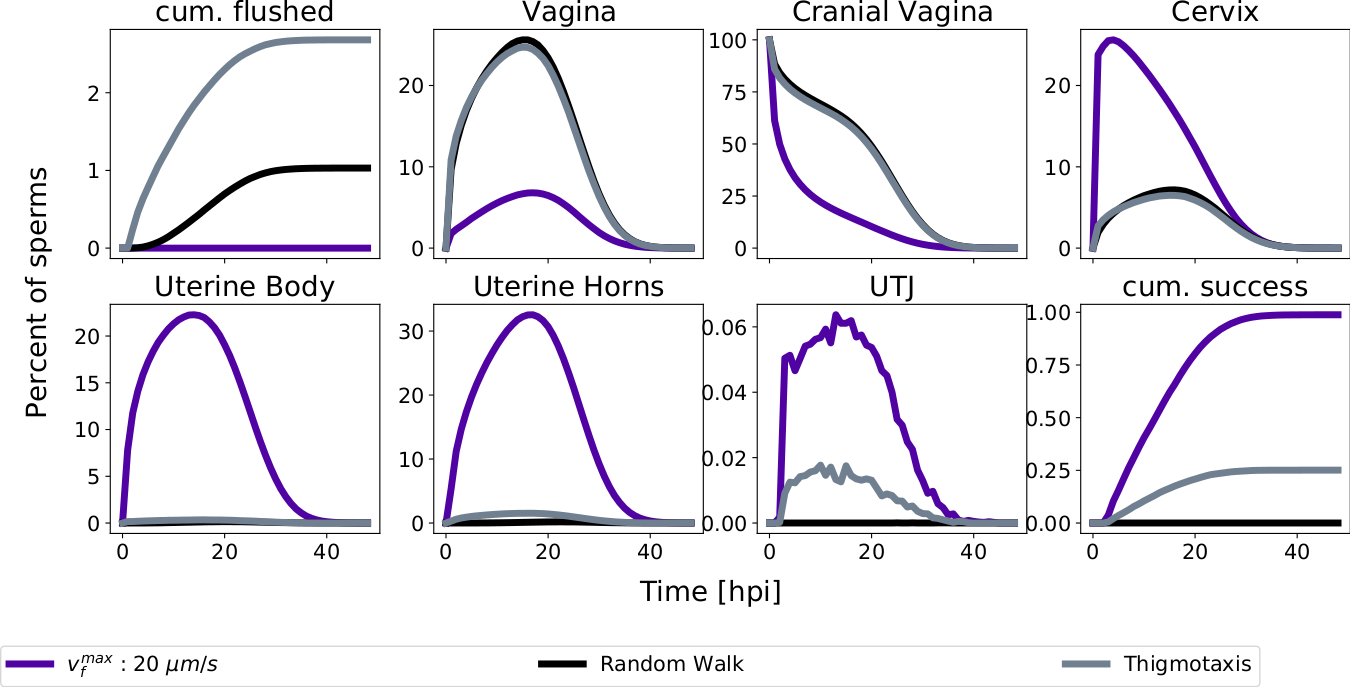

Supplement: S11 Fig — Thigmotaxis alone helps sperms to reach the oviduct. Under optimal settings of fluid flow up to 1% of sperms surpass the UTJ. (TIF) [file pcbi.1009109.s016.tif]

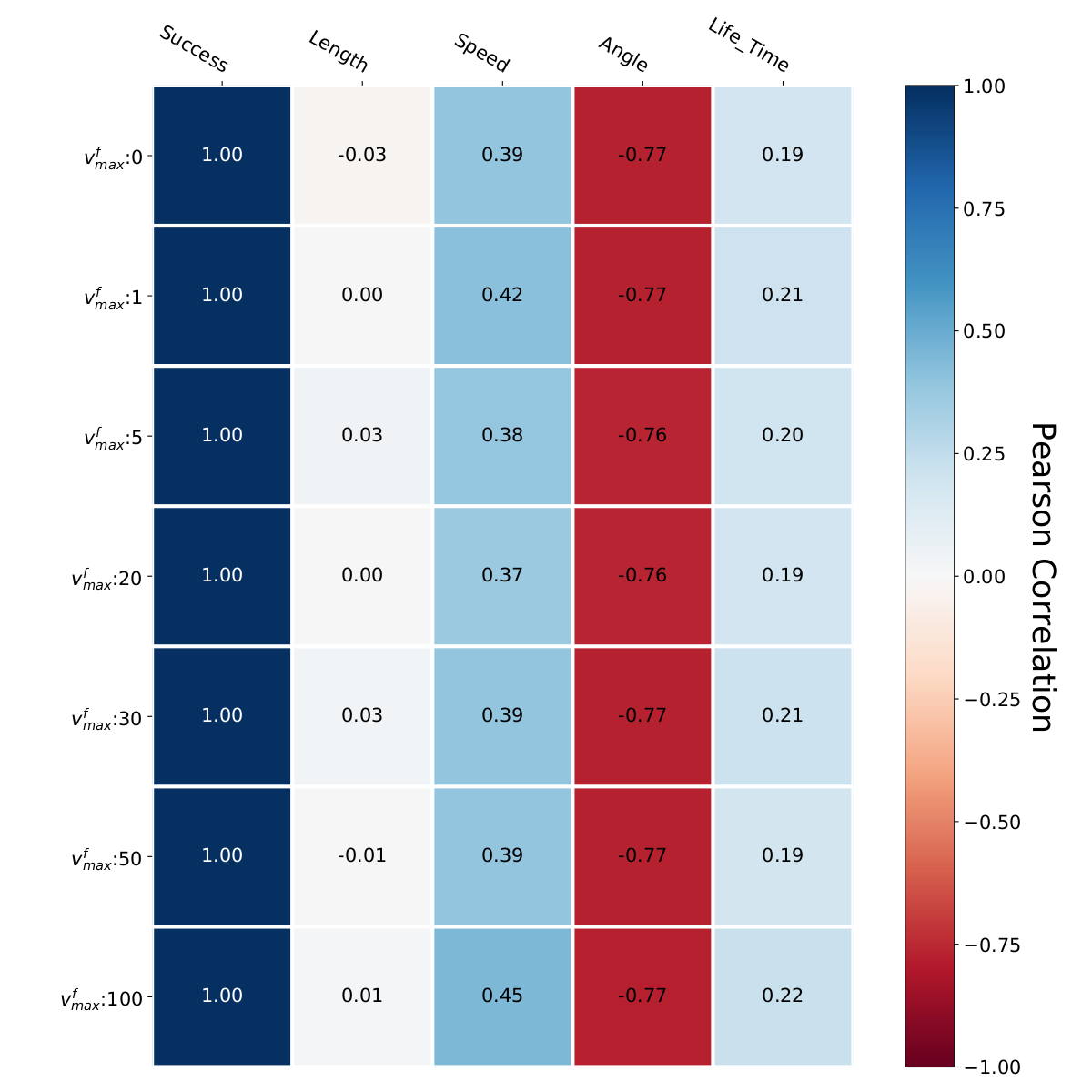

Supplement: S12 Fig — Pearson correlation between success and sperm properties for different fluid velocities. The speed of sperm correlates positive with success, while a less persistent movement (large deflection angle) correlates negatively with success. Sperm life time shows a small positive correlation with success. Sperm length does not correlate with success in the model. (TIF) [file pcbi.1009109.s017.tif]

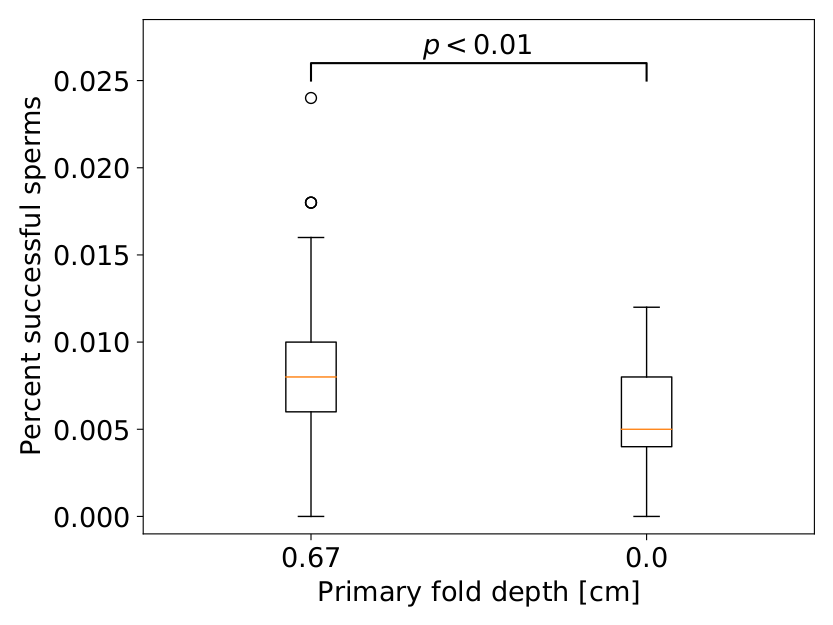

Supplement: S13 Fig — Agents are significantly more successful in geometry with primary and secondary folds. Those simulations were performed with no fluid flow. P-value was calculated with a two-sided t-test from the python scipy package [50]. The Null hypothesis was that both settings have an equal expected value. (TIF) [file pcbi.1009109.s018.tif]
