# Supplementary material for: Sperm migration in the genital tract—In silico experiments identify key factors for reproductive success
Source: PLoS Comput Biol. 2021 Jul 15;17(7):e1009109. doi: 10.1371/journal.pcbi.1009109 (PMC8282070; doi:10.1371/journal.pcbi.1009109)
Supplement: Attachment — Submitted filename: Reproducible_report_PCOMPBIOL_D_20_01294.pdf [file pcbi.1009109.s020.pdf]

Reproducibility report for: Sperm migration in the genital tract - in silico experiments identify key factors for reproductive success.
Submitted to: PLoS Computational Biology
Manuscript number/identifier: PCOMPBIOL-D-20-01294

Curation outcome summary: Unable to determine the reproducibility of the work presented.

Box 1: Criteria for repeatability and reproducibility

- ☐ **Model source code provided:**
  - ☐ Source code: a standard procedural language is used (e.g. MATLAB, Python, C)
    - ☐ There are details/documentation on how the source code was compiled
    - ☐ There are details on how to run the code in the provided documentation
    - ☐ The initial conditions are provided for each of the simulations
    - ☐ Details for creating reported graphical results from the simulation results
  - ☐ Source code: a declarative language is used (e.g. SBML, CellML, NeuroML)
    - ☐ The algorithms used are defined or cited in previous articles
    - ☐ The algorithm parameters are defined
    - ☐ Post-processing of the results are described in sufficient detail
- ☐ **Executable model provided:**
  - ☐ The model is executable without source (e.g. desktop application, compiled code, online service)
    - ☐ There are sufficient details to repeat the required simulation experiments
- ■ **The model is described mathematically in the article(s):**
  - ■ Equations representing the biological system
  - ■ There are tables or lists of parameter values
  - ☐ There are tables or lists of initial conditions
  - ☐ Machine-readable tables of parameter values
  - ☐ Machine-readable tables of initial conditions
- ☐ **The simulation experiments using the model are described mathematically in the article:**
  - ☐ Integration algorithms used are defined
  - ☐ Stochastic algorithms used are defined
  - ☐ Random number generator algorithms used are defined
  - ☐ Parameter fitting algorithms are defined
  - ☐ The paper indicates how the algorithms yield the desired output

Box 2: Criteria for accessibility

- ☐ Model/source code is available at a public repository or researcher's web site
  - ☐ Prohibitive license provided
  - ☐ Permissive license provided
  - ☐ Open-source license provided
- ☐ All initial conditions and parameters are provided
- ☐ All simulation experiments are fully defined (events listed, collection times and measurements specified, algorithms provided, simulator specified, etc.)

Box 3: Rules for Credible practice of Modeling and Simulation[a]

[a]Model credibility is assessed using the Interagency Modeling and Ananlysis Group conformance rubric: https://www.imagwiki.nibib.nih.gov/content/10-simple-rules-conformance-rubric

- ■ Define context clearly: Extensive
- ■ Use appropriate data: Extensive
- ■ Evaluate within context: Extensive
- ☐ List limitations explicitly: Insufficient
- ☐ Use version control: Insufficient
- ■ Document adequately: Extensive
- ☐ Conform to standards: Insufficient

Box 4: Evaluation

- ☐ Model and its simulations could be repeated using provided declarative or procedural code
- ☐ Model and its simulations could be reproduced

Summary comments: No model source code or data was submitted for download as a zip file, Github link or in any other format. As a result we are unable to determine the reproducibility of the work presented.

Anand K. Rampadarath[1], PhD
Curator
Center for Reproducible Biomedical Modeling

David P. Nickerson, PhD
Curation Service Director
Center for Reproducible Biomedical Modeling

Auckland Bioengineering Institute,
University of Auckland

[1]Email: a.rampadarath@auckland.ac.nz
