# Supplementary material for: Sperm migration in the genital tract—In silico experiments identify key factors for reproductive success
Source: PLoS Comput Biol. 2021 Jul 15;17(7):e1009109. doi: 10.1371/journal.pcbi.1009109 (PMC8282070; doi:10.1371/journal.pcbi.1009109)
Supplement: Attachment — Submitted filename: Response_letter_PCOMPBIOL-D-20-01294.pdf [file pcbi.1009109.s021.pdf]

Ref: PCOMPBIOL-D-20-01294 **Berlin, 21 December 2020**

Dear Drs. Finley and Papin,

Thank you for the first evaluation of our manuscript:

Sperm migration in the genital tract - in silico experiments identify key factors for reproductive success

by Jorin Diemer, Jens Hahn, Björn Goldenbogen, Karin Müller and Edda Klipp.

We also thank the reviewers for their suggestions and precise remarks that allowed us to essentially revise our manuscript.

Please, find below our detailed point-by-point responses to the concerns and suggestions of the reviewers (***bold italic letters***).

Comments to the Authors:

***Reviewer #1:* The Reproducibility report has been uploaded as an attachment.**

The complete Python model code is available in a public git repository at

https://ford.biologie.hu-berlin.de/jorin/female_sperm_selection

under the GNU *General Public License*, version *3*. The repository contains all files necessary to simulate the model and reproduce the presented figures. Additionally, a yml file is provided to set up a conda virtual environment to avoid complications due to package dependencies when running the model.

***Reviewer #2:* This is a high-quality manuscript presenting a comprehensive analysis of sperm migration in a realistic 3D geometry of the female genital tract,**

accounting for the most important biophysical processes: thigmotaxis, rheotaxis, immune activity.

The authors report several robust qualitative findings (using bovine fertilization as model system), namely:

- without thigmotaxis, virtually zero sperm reach the fertilization site

- with thigmotaxis, but no rheotaxis, at least 0.01% of deposited sperm would reach the fertilization site

- with thigmotaxis and rheotaxis, this number increases 10-fold to 0.1%

- migration through the reproductive tract selects for faster sperm cells with less directional fluctuations

This manuscript addresses an important topic of both academic and medical relevance,

and should appeal to a broad interdisciplinary readership,

including biologists and clinicians interested in assisted fertilization, and theoretical biological physicists, especially those with an interest in biological fluid dynamics.

The mathematical model is well-crafted and to the best of my knowledge the reported findings are novel.

The manuscript is well-written with a clear structure and exposition of results.

Clear figures explain modeling assumptions in visual form.

The introduction provides a lucid motivation for the study (discussing assisted reproduction and conservation efforts for endangered species).

While the manuscript may be in principle suitable for PLoS Computational Biology,

I want to suggest that the authors address the following comments.

We thank the reviewer for their very positive and encouraging evaluation of our work! We also thank them for the effort and time spent to improve the manuscript. We tried our best to address the following comments and hope that the revised version might be agreed to become published.

Major comments

1.

I feel the manuscript would strongly benefit if the authors could relate their simple phenomenological descriptions of thigmotaxis and rheotaxis to previously published detailed models.

Ideally, this would provide further justification for the simple phenomenological Ansatzes for thigmotaxis and rheotaxis used e.g. in Eqs. (S28) and (S47).

Similarly, effective parameters in the current model could be derived as "best fits" to previously reported detailed models.

There exists a number of detailed theoretical models for thigmotaxis and rheotaxis of individual swimmers in the literature:

- For thigmotaxis, e.g. both Jens Elgeti & Gerhard Gompper as well as David Smith & Kirkman-Brown published several papers on boundary alignment of swimming sperm cells. In particular, Elgeti et al. compared these detailed models to a minimal model of a self-propelled cylinder interacting with a boundary wall, discussing in detail the confined rotational diffusion of the swimmer's orientation vector [Elgeti et al. EPJ-ST 2016].

- For rheotaxis, Kantsler et al., proposed the effective 'weather-vane model' to explain the anti-alignment of actively swimming sperm cells with external flow [Kantsler, Goldstein et al. eLife, 2014]. While this might have been the first theory paper on rheotaxis after the experimental findings by Miki et al., there are many more theory papers that followed thereafter (which can be found among the 271 citations of Miki et al. as of today)

I urge the authors to compare their minimal models to these previous more detailed models.

This will provide a crucial justification for their minimal models used in their study.

In order to justify our minimal models, we compared our model of thigmotaxis with data published by Nosrati et al. (2016) (https://www.nature.com/articles/srep26669), while the rheotaxis model was compared to data and model from Kantsler et al. (2014) (https://elifesciences.org/articles/02403).

Thigmotaxis:

Nosrati et al. recorded sperm x and y positions in channels with different widths. They found an increased sperm concentration near walls and in channel corners. Depending on this channel width different percentages of corner, wall and bulk swimmers were identified. Our model reproduces the accumulation of sperms in corners and at walls. The results are included in Figure 2G and H. As the percentage of corner and wall swimmers are slightly underestimated, the effect of thigmotaxis in our model is likely also underestimated.

We included the following statement in the section **Box model reproduces *in vitro* dynamics of sperm:**

"Nosrati et al. (31) measured bull sperm densities in channels of varying width (50µm,100µm and 400µm). Depending on their distance to channel wall and corners sperms were classified as wall, corner or bulk swimmer. With increasing channel size the percentage of corner swimmers decreased from approximately 80% to around 30%. The presented model predicts a decrease from approximately 60% to around 10% (Fig. 2G, H). Hydrodynamic properties determining motion and orientation of sperms at surfaces are addressed in more advanced models (29, 32), while our minimal model is based on vector additions when agents are in proximity of the compartment boundary. In contrast to the advanced models our sperms are not attracted towards the wall over long distances.This potentially leads to the slightly lower percentage of agents classified as corner and wall swimmers Fig. 2G and H, compared with data

from Nosrati et al. (31). As a result, the effect of thigmotaxis is likely underestimated. However,the agents tend to swim along surfaces as shown in Fig. 2E."

Comparing the thigmotaxis model with data from Nosrati et al. shows that we slightly underestimate this effect. While Nosrati et al. report that 80 % of sperms are corner swimmers in a channel with 50 µm width, our model predicts around 60 %. This is most likely due to missing long distance attraction between boundaries and sperms, which is included in more detailed models for thigmotaxis (Elgeti & Gompper 2016, https://link.springer.com/article/10.1140/epjst/e2016-60070-6).

Rheotaxis:

Kantsler et al. (2014) tracked bull sperm positions in a microfluidic device during flow reversal. Further they present a sophisticated model of sperm rheotaxis which includes fluid viscosity. A feature our description is missing. Depending on the fluid viscosity Kantsler er al. measured a reorientation time of 5s (at 1mPas) to 50s (at 12 mPas, which is roughly 4x higher than the viscosity of natural ejaculate). Our model predicts a reorientation time around 20s and thereby perfectly agrees with the data from Kantsler et al. The results are included in Figure 2I.

We included the following statement in the section **Box model reproduces *in vitro* dynamics of sperm:**

"To justify the outcome of our minimal model in comparison with a more detailed model by Kantsler et al. (23), we tested the behavior of our agents upon fluid flow reversal. In agreement with the referred study, after an initial peak in the upstream velocity, agents re-orientate within 20s (Fig. 2I)."

2.

As will most mathematical models of a certain level of detail, this model comprises a large number of parameters.

I appreciate that parameters are presented in tabulated form and references are provided wherever possible.

Nonetheless, a sensitivity analysis would substantially strengthen the reported results.

- What are the key parameters that influence fertilization success most?

- Would a simpler model, say without the undulating surface of the reproductive tract, yield similar results?

- What role does the variability of flagellar length play?

A full sensitivity analysis is hard to perform due to the large number of parameters and the computationally expensive nature of the simulations. However, as pointed out in the main text,

especially a fluid flow as well as the agent properties of deflection angle and speed influence sperm success (to reach to oviduct). In the model, the agents recognize the compartment boundary earlier when they are longer. Thus, in the case of thigmotaxis they would start to align earlier. However, we calculated the Pearson correlation between sperm properties and being successful, see Fig.S12. There was no correlation between the length of an agent and the sperm success.

We added the following statement to the **Discussion**:

"Whereas the sperm length in its current parameter mode has no significant impact on sperm success (Fig. S12), the length of the principle plus terminal piece of the flagellum in relation to the midpiece seems to be physiologically related to the swimming speed of mammalian sperm as well as the straightness of motion(42). Therefore, our minimal model would explain experimental data on the observed higher fertility of males with faster sperm."

To investigate the effect of the undulated surface we performed a simulation without folds and without fluid flow and compared the results to the simulation with folds. The results are shown in Figure S13. The enhanced surface area seems to have an effect on the success rate of sperms.

We added the following statement to the section **Wall alignment facilitates directed motion**.

"Additionally, we simulated agents in a geometry without primary and secondary folds, showing that a wrinkled surface enhanced the effect of thigmotaxis, as the success rate without folds was 0.00545% (Fig. S13)".

We replaced the following passage with the new version in the **Discussion**:

"By approximating the hydrodynamic properties of sperm, the percentage of agents reaching the oviduct already rose to 0.0084%, which lies in the range of 0.0001% to 0.1% reported by Eisenbach et al.(11) and Reynaud et al.(2), respectively."

"By approximating the hydrodynamic properties of sperm, the percentage of agents reaching the oviduct already rose to 0.0084%(0.0055% without primary and secondary folds, Fig. S13). The increase in successful agents in the presence of folds indicates that microgrooves might be beneficial for sperms due to the larger surface area. The aforementioned success rates lie within the range of 0.0001% to 0.1% reported by Eisenbach et al.(11) and Reynaud et al.(2), respectively. "

Medium comments

3a.

How would results change for longer search times, or in the absence of immune activity?

3b.

The authors show that thigmotaxis improves fertilization success.

But it is not directly clear if this is a direct consequence of this motility behavior,

or if thigmotaxis helps sperm cells to hide from immune responses inside the grooves of the reproductive tract.

Simulations of thigmotaxis in the absence of immune activity should answer this question.

To address comments 3a and 3b we simulated the model for 48 hours with three different settings, all of them without the removal due to the immune system. First, we simulated sperms (N=10^6) without thigmotaxis and fluid flow (persistent random walk) and found that no agent was successful. Adding thigmotaxis led to 0.25% successful agents, while an additional fluid flow of 20 μm/s increased the percentage of successful agents to 1%. The results are shown in Figure S11.

We included the following statement in the section **Wall alignment facilitates directed motion:**

"To investigate whether the increased success of agents is directly linked to thigmotaxis or if it is a secondary effect due to evasion of the immune system, we simulated long term experiments (48 h) without immune system, but with and without thigmotaxis. The results show that even for long simulation times no agent reaches the oviduct without thigmotaxis, while wall alignment boosts success to 0.25%, Fig. S11."

We included the following statement in the section **Fluid flow aligns sperm motion and boosts their success:**

"To estimate the maximal percentage of successful sperms we simulated agents in a tract without immune system and a fluid velocity of 20μms and found that up to1% of agents are successful without removal by immune responses (Fig. S11)."

We included the following statement in the section **Supplementary Note 5: Thigmotaxis aids transition through cervix and UTJ:**

"To investigate if the positive effect of thigmotaxis is a direct consequence of wall alignment or if thigmotaxis helps the agents to evade the immune system by hiding in microgrooves we performed simulations without immune system and a simulation time of 48h, Fig. S11. As no agent reaches the oviducts without thigmotaxis, but≈0.25% do with thigmotaxis, this motion characteristic is responsible for the higher number of successful agents."

3c.

How were the parameters for modeling the immune response determined?

The largest part of sperm reaches the UTJ/oviduct in most of the studied species within several hours after mating. And with the exception of long-term sperm storage (e. g. in female bats) sperm of most mammalian species may survive about two days (maximum of five days) in an appropriate environment such as oviduct. Therefore, the mean sperm survival in the lower parts of the genital tract was initially set to 24 +/- 6 hours. As explained in the manuscript, we also refer to observations that the female immune response (e. g. infiltration of vagina, cervix, uterus with leukocytes) develops gradually within the first hours after mating [7, 29]. Consequently, a progressively increasing reduction of lifetime with time was chosen. This is a first simple approximation of the far more complicated immune response which might be further developed in future, for instance by introducing protective components, encounters with agglutinating agents (e. g. NET) etc..

We included the following statement in the section **Survival rate is modulated by immune system:**

"According to the observation that sperm of most mammalian species survive in the oviduct for a maximum of five days after mating (37,38). Therefore, our agents in the less sperm-friendly lower part of the female genital tract have a lifetime drawn from a normal distribution around 24h (Tab. 1)."

4a.

For their rheotaxis model, the authors assume that flows starts below the UTJ compartment,

but that there is no flow in the oviduct itself (page 12, B, bullet point 1).

However, Miki et al. reported flows in the oviducts of mice, and proposed that these induce a relevant rheotaxis response

[Ref. 30: Miki & Clapham, Curr. Biol., 2013].

The authors should discuss this discrepancy.

As the oviduct itself is not considered in our model, the bullet point is indeed misleading. For simplicity, we assumed no fluid flow through the UTJ. However, as there is a fluid flow in the oviducts, testing different fluid velocities through the UTJ will be of interest for future simulations. As the oviduct itself is not part of the model and there is a fluid flow within the oviducts we changed the bullet point, it now reads:

“No flow through the UTJ“

Further we included the following statement in the **Discussion**:

“In the present model we did not consider a fluid flow from this compartment but it could be asked in future studies whether such a fluid flow could act as physical and/or chemical signal to guide the sperm into the oviduct.”

This sentence is preceded by a sentence about the UTJ (see answer to 4b). Hence “this compartment” refers to the UTJ.

4b.

For typical flow speeds in the reproductive tract, the authors cite Miki et al., who measured 20um/s in murine oviducts.

However, the radius of the oviduct is much smaller than the radii of the uterine cavity or cervix.

Thus, assuming the same flow speed in these compartments as in the oviduct would actually correspond to a substantially larger total volume flow.

Can the authors justify why the flow speeds considered represent the physiological range?

Actually, we have no experimental proof that the optimum flow speed in our model is in a physiological range in the lower part of the bovine female tract. We only have the experimental values for the conditions in the oviduct reported by Miki et al.. However, it is known that a remarkable outflow of secretions occurs from the bovine vagina after intercourse. The volume of bovine cervical mucus secreted at oestrus can reach up to 100 mL per day [Cortés ME, González F, Vigil P. Crystallization of Bovine Cervical Mucus at Oestrus: An Update. Rev Med Vet. 2014; (28): 103-16.].

Fig. S6A shows the volume flow profile for a maximal fluid velocity of 50 μm/s (Fig S6D). The outflow would be around 350 μl/min (100ml / 285 minutes). This would mean that a cow in oestrus would have to replace its cervical mucus appr. every 4-5h. We could not find any data on mucus production rates in bovine females. With the maximal flow velocity which results in the largest fraction of agents reaching the oviduct (20 μm/s), the outflow would lie around 140 μl/s (~100ml / 12h). Therefore, we think the considered flow speeds are at least in a physiological order of magnitude.

We included the following statement in the **Discussion**:

“However, depending on the fluid velocity a certain volume of fluid has to be expressed in the system. A maximal fluid velocity of 20 µm/s corresponds to a mucus volume ’leaving’ the system of 2.44 µL/s. This would result in a volume of 211 mL/day. Bovine cervical fluid can reach a volume of 100 mL/day (40), which would throughout correspond to the predicted value after simulation. Further, it might be considered that contractions of the female reproductive tract (41) can locally reveal high fluid velocities with smaller volumes of secretions. This could also be valid for the UTJ where the diameter reduction would accelerate the oviduct secretions. ”

5.

The authors mention additional biophysical processes in the discussion, but stay very brief on this.

I feel the manuscript would benefit if this section would be extended and references to the relevant literature were added.

- For example, what is the supposed role of progesterone-induced motilty responses?

Will these play a role only in the oviduct, or also in other compartments of the reproductive tract?

Can the authors review the state of the field whether progesterone induces chemokinesis or chemotaxis?

- Sperm cells interact with the ciliated epithelium in the oviduct:

To what extent is this interaction captured by the corrugated fine structure of the oviduct surface with primary and secondary folds as used here?

- The cervix is visco-elastic, while all fluids are treated as Newtonian fluids in this study:

Can the authors predict how the reported results would change qualitatively in a non-Newtonian fluid?

This question is addressed in **Supplement Note 2B Paragraph Poiseuille Profile**.

“For Non-Newtonian fluids the decrease in fluid velocity towards the boundaries becomes steeper (50), keeping the general appearance of the profile. Therefore the positive effect of positive rheotaxis might even be underestimated.”

- Is it known at which stage of a sperm cell's progression through the reproduction tract capacitation occurs?

When do sperm cells become hyperactivated?

- Penetration of the cumulus layer represents an additional obstacle for fertilization that should be mentioned at least.

- Thermotaxis is a concept that might need discussion: there seems to be some indirect evidence from the Eisenbach laboratory but no firm confirmation and certainly no consensus in the field.

Even if the present study captures only the migration to the oviduct, and does not yet account for additional mechanisms inside the oviduct,

(which are insufficiently understood at present) , this study represents an important contribution that should be published.

However, as a service to the reader, this manuscript should connect to the current state of the field.

We agree that there exists a bunch of biophysical and biochemical processes to become considered in a model. However, as stated above, we did not intend to include the sperm behaviour in the oviduct in the presented study because it is still and first a challenge in many mammalian species to understand reproductive failures of sperms on their way to the oviduct. This is particularly true if artificial insemination is needed as it is in human and endangered animals or animal breeding.

Progesterone may act as chemoattractant and/or may have a signalling effect on motion characteristics dedicated to capacitation. Both probably occur in the near vicinity of the egg. Thermotaxis is still under debate. However, we think that a review on chemokinesis, chemotaxis, thermotaxis would be beyond the scope of the study. Therefore we decided to restrict this part of outlook to the keywords, which are surely familiar to the more specialized reader.

We included the following statement in the **Discussion**:

"The complex processes occurring to sperm in the oviduct are a further topic to extend/complete the simulation of sperm migration to the oocyte. Chemotaxis, thermotaxis, and rheotaxis as well as sperm interactions with epithelial cells and capacitation-related metabolic changes leading to hyperactivation of sperm motion are currently regarded and investigated as key processes prior to fertilization (13, 33, 43)."

6.

The authors calibrate the 'standard deviation of the deflection angle' in their model,

by comparing simulated trajectories to a previously measured straightness parameter STR.

However, it makes a difference whether such a comparison is done for 2D- or 3D-trajectories.

Is it correct that the previous experimental data by Tung et al. reports on effectively two-dimensional sperm trajectories in a shallow observation chamber

[Ref. 18: Tung, Suarez et al. PNAS 2015]?

If the authors likewise simulated 2D-trajectories whose statistics matched those of the experimental trajectories,

then the 'standard deviation of the deflection angle' should be multiplied by a factor 'sqrt(2)' if I am not mistaken.

I ask the authors to give attention to this technical point.

In order to compare the agent persistence to the persistence measured by Tung et al. we simulated agents in a shallow 3D chamber (120µm height, as the microfluidic used by Tung et al.). In order to calculate agent persistence we divided the distance from start to end point by the total distance travelled. Thus we included distances traveled in z direction in our calculation. We recalculated persistence by neglecting the z direction in order to directly compare it to Tung et al.. We actually found two errors due to your comment. First, in the caption of Figure S9 we accidently wrote 20µm instead of 120 µm and second we simulated in a box of height 200 µm. Thus we repeated the simulations in a box of height 120 µm. The figure below shows the newly calculated persistence, neglecting the z direction. It is still in agreement with Tung et al. (0.87 +-2). Logically, we updated the caption of Figure S9 as well as the figure itself.

We also removed the sentence “With a height of 20µm, the box represents a typical specimen chamber (23). ” from **Supplementary Note 4** and added “of height 120µm” to specify the dimension of the chamber used by Tung et al.

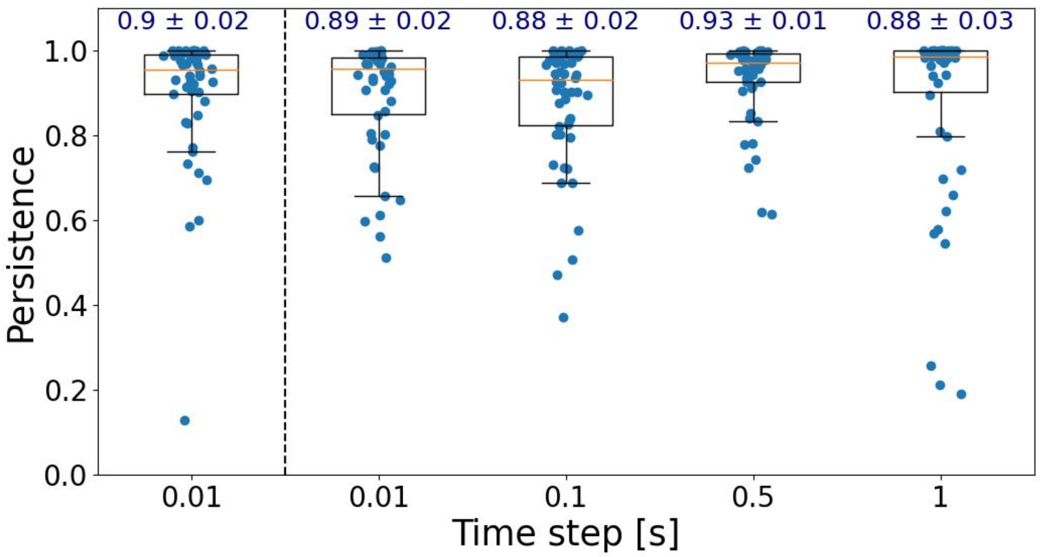

7.

The authors describe sperm motion as a three-dimensional persistent random walk with fluctuationg speed, which seems an admissable first approximation.

As a service to the reader, the authors should relate this model to similar models such as persistence random walks, Active Brownian Particles, correlated random walks, etc., all of which embody essentially the same idea.

With a little bit of work, I think the authors may even find an analytical formula that links the three analogous concepts

- STR (for a given trajectory length; called 'persistence' in Ref. 18: Tung et al. PNAS 2015)

- 'standard deviation of the deflection angle'

- persistence length

We included the following statement in the section **Temporal model of sperm movement**.

Thus, sperm movement without any interaction is a spatially restricted, "...unbiased persistent (correlated) random walk, i.e. that an agent's orientation depends on its former orientation and has no directional bias (as the mean deflection angle is 0). This is a widespread concept to describe self-propelling particles similar to active Brownian motion and widely used in biological models(21, 22)."

The persistence length or degree of correlation is given by the average of the cosine of the deflection angle (turning angle), which is the finite integral from -π to π of the cosine time the probability distribution of the deflection angle [Edward A. Codling, Michael J. Plank, and Simon

Benhamou. Random walk models in biology, aug 2008. ISSN 17425662.][P. Romanczuk, M. Bär, W. Ebeling, B. Lindner, and L. Schimansky-Geier. Active Brownian Particles: From individual to collective stochastic dynamics: From individual to collective stochastic dynamics, mar 2012. ISSN 19516355].
Hence, the persistence length can be calculated from the 'standard deviation of the deflection angle' (Θ_s^{SD}) by:

$$\begin{aligned}\overline{cos(\Theta_s)} &= \int_{-\pi}^{\pi} cos(\Theta_s)\mathcal{P}(\Theta_s)d\Theta_s \\ &= \int_{-\pi}^{\pi} cos(\Theta_s)\mathcal{N}(0,\Theta_s^{SD})d\Theta_s\end{aligned}$$

Where *P* denotes the probability distribution of Θ_s and *N* the normal distribution with mean 0 and standard deviation Θ_s^{SD} .

8.

While the model is truely three-dimensional, a casual reader may actually miss this point.

For example, Fig. 2 and Fig. 3 give the visual impression of a 2D model.

Also, the description of persistent sperm motion of 2D reads like the description of a two-dimensional persistent random walk - only in the Methods section does it become clear that the authors consider a three-dimensional persistent random walk.

To emphasize that we actually describe a three dimensional movement we included the following statement in the section **Temporal model of sperm movement**.

"Subsequently, the deflected vector is turned around the original orientation by an angle uniformly distributed between -π and π. This results in truly three-dimensional movement within the reconstructed female genital tract."

9.

While discussing Poiseuille flow, the authors write "speed increases quadratically with distance" [introduction & p. 12]

whereas 'u(d) ~ d_max^2 - (d_max-d)^2', see also Eq. (S40).

I find the formulation at least unclear, as it seems to wrongly suggest 'u(d) ~ d^2'.

As d(x,y) represents the distance to the wall, while d_max represents the maximal distance from wall to the compartment center. Therefore the flux velocity is maximal in the center (d(x,y=d_max)) and 0 at the boundaries (d(x,y) = 0).

10a.

I did not immediatedly see why Eq. (S28) ensures time-step independent alignment.

A short derivation or reference would be appreciated.

Eq . S28 takes the weighted average of two vectors (sperm direction and its normalized projection on the wall). The reasoning is that, given two vectors within a 1 second the angle between those is halfened. In the next timestep the updated sperm direction vector is again averaged with its projection on the wall. Within 2 seconds the angle should be halfened two times. Under the assumption that the direction of the projection vector does not change (which should be the case, as the sperm itself is magnitudes smaller than the female genital tract), one can also take the average between the (fixed) projection vector and the (variable) sperm direction vector.

Given two vector $\vec{a}$ and $\vec{b}$, where $\vec{a}$ is fixed and $\vec{b}$ is variable, i.e. representing projection vector and sperm direction respectively. Taking the average ones gives the vector $\vec{b_{t+1}}$. To obtain vector $\vec{b_{t+2}}$ we have to average $\vec{b_{t+1}}$ with the fixed vector $\vec{a}$. Thus $\vec{b_{t+2}} = (3\vec{a_t} + \vec{b_t}) / 4$. See below:

$$f(n) = \frac{(2^n - 1)\vec{a}_t + \vec{b}_t}{2^n}$$

$$\vec{b}_{t+1} = f(n=1) = \frac{\vec{a}_t + \vec{b}_t}{2}$$

$$\vec{b}_{t+2} = f(n=2) = \frac{f(n=1) + \vec{a}_t}{2} = \frac{\vec{b}_{t+1} + \vec{a}}{2} = \frac{\frac{\vec{a}_t + \vec{b}_t}{2} + \vec{a}_t}{2}$$

$$= \frac{3\vec{a}_t + \vec{b}_t}{4}$$

We can show that the function f(n) (Eq. S28) calculates the weighted average keeping vector $\vec{a}$ fixed with mathematical induction, taking f(n=1) and f(n=2) as induction anchor. The step from n to n+1 is shown below:

$$\vec{b}_{t+n+1} = \frac{\vec{b}_{t+n} + \vec{a}}{2} = \frac{f(n) + \vec{a}}{2} = \frac{\frac{(2^n - 1)\vec{a} + \vec{b}}{2^n} + \vec{a}}{2}$$

$$= \frac{(2^n - 1)\vec{a} + \vec{b} + 2^n\vec{a}}{2^n \cdot 2}$$

$$= \frac{\vec{a}(2^n - 1 + 2^n) + \vec{b}}{2^{n+1}}$$

$$= \frac{\vec{a}(2 \cdot 2^n - 1) + \vec{b}}{2^{n+1}}$$

$$= \frac{\vec{a}(2^{n+1} - 1) + \vec{b}}{2^{n+1}} = f(n+1) \quad \textbf{q.e.d.}$$

Thus Eq. S28 takes the weighted average such that it calculates repeated vector averages, changing the sperm direction (here $\vec{b}$) while keeping the projection on the wall fixed (here $\vec{a}$).

However, the equation neglects that the sperm direction vector is normalized in each time step. Thus taking larger timesteps, the sperms direction vector is normalized less often and the formula overestimated the alignment (as $\vec{b_{t+1}}$ might be shorter than 1 but vector $\vec{a}$ has length 1).

To estimate the error introduced by this neglection, we took two vectors with varying initial angles between them and calculated the resulting vector from the formula taking the weighted average and by repeatedly averaging and normalizing the vectors for n=dt times. We than calculated the remaining angle between the resulting vector (respresting the updated sperm direction or $\vec{b_{t+n}}$) and the fixed projection vector $\vec{a}$. The remaining angle for different initial angles and different ns (dts) is shown below. Solid lines show the remaining angle after repeated averaging and normalizing. Dashed lines show remaining angles using the formula.

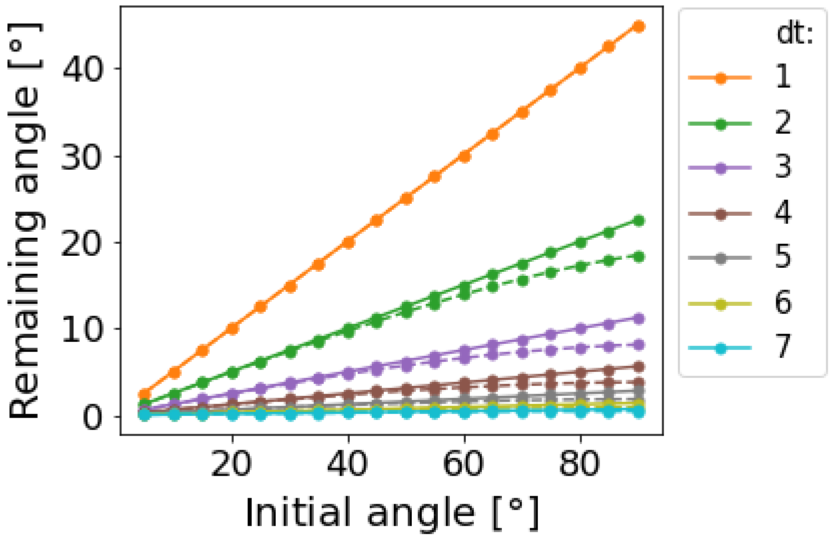

Next we calculated the difference between the solid ans dashed lines to get the error introduced by Eq. S28. The maximal error occurs at dt=2 and initial angle =90° and is just above 4°.

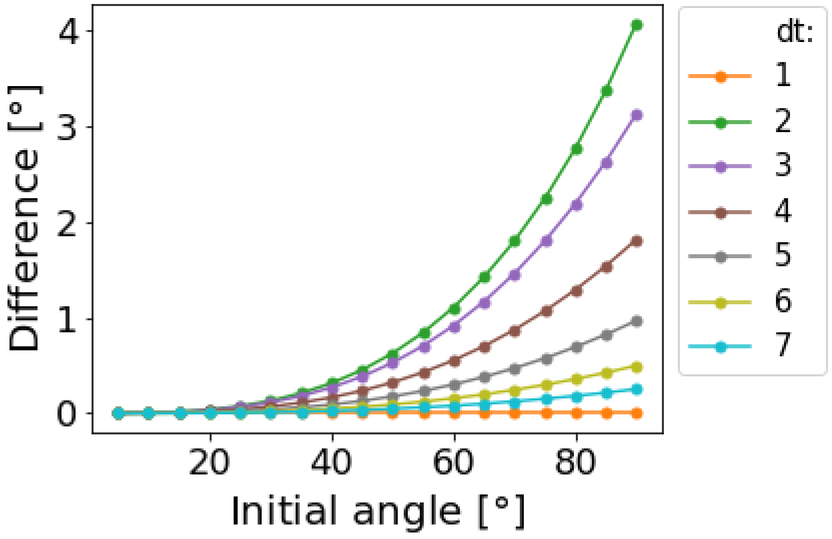

The derivation above only works for dt >= 1. For dt < 1 we estimated the possible error as follows: Given a small time step we calculated how often Eq S28 would be applied until 1 second would be over (e.g. for dt = 0.1, n = 1 /dt = 10 times). Than we applied Eq S28 n times and compared the resulting angle to the angle we would expect when turning once with dt = 1.

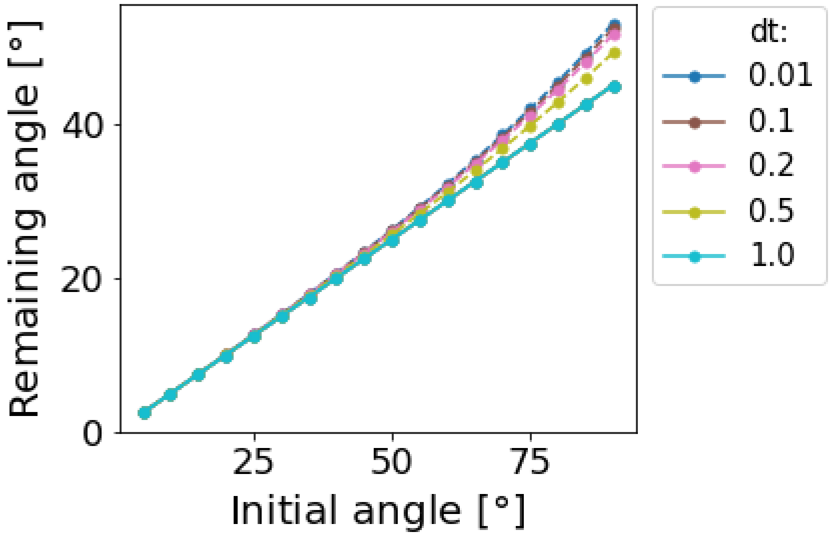

This figure shows the remaining angle between the fixed vector and the turned sperm orientation vectors, while the angle for dt = 1 is the desired vector. Thus, using small timesteps slightly lowers the strength of wall alignment (as the remaining angle is larger than for dt = 1). The difference is plotted below:

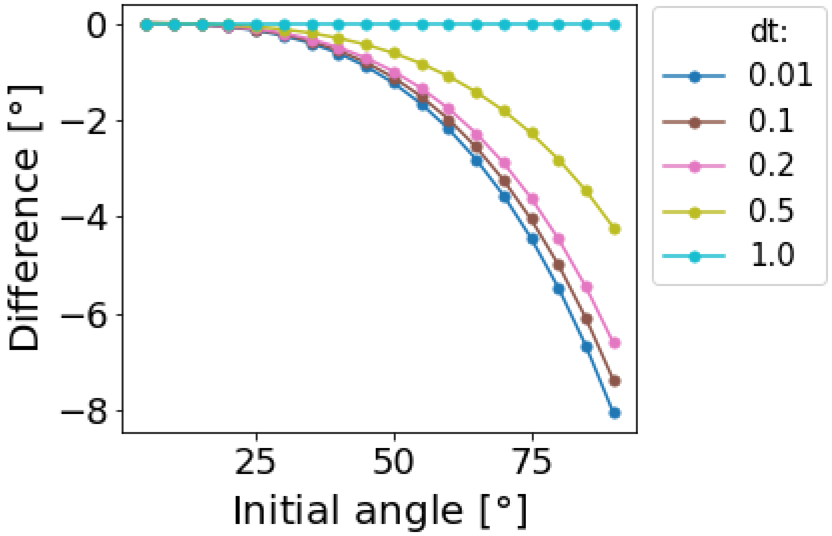

Thus for a time step of dt=0.1, the sperm orientation vector would be turned 7° less than expected for dt=1 with an initial angle of 90°.

We want to note that we always used dt=1 except for the simulations in the box which we used to compare our data to the data from Nosrati et al. (Fig.2G,H). We thank a lot for pointing out this inaccuracy.

We replaced “The term 2^dt ensures...” with “The term 2^dt approximates...” in **Supplementary Note 2A.**

10b.

For a physicist, an expression like '2^dt' as in Eq. (S28) is not defined because

the time-step 'dt' has physical units of a time and can thus not used as an exponent.

The authors should state clearly how '2^dt' should be interpreted

[even if it is just '2^(dt / 1 second)' because '2^(dt / 1 year)' would be equally fine].

We changed Eq. S28 such that it reads “2^(dt / 1 second)” instead of “2^(dt)”.

11.

The Ansatz for the microstructure of the compartments Eq. (S2)-(S24) contains many unknown parameters.

The authors should explain in more detail how they achieved their "educated guesses" for these parameters (listed in Table S1).

Could these parameters probably be estimated from microscope images?

We estimated those parameters on the microscopic images provided by Mullins and Saacke [K. June Mullins and R. G. Saacke. Study of the functional anatomy of bovine cervical mucosa with special reference to mucus secretion and sperm transport. The Anatomical Record, 225(2):106–117, oct 1989. ISSN 10970185. doi: 10.1002/ar.1092250205.].

We added “Educated guesses for the number of microgrooves were taken with the help of microscopic images and sketches from Mullins and Saacke” to the caption of Table S1.

12.

I suppose Eq. (S46) contains a typo, because a scalar on the left-hand side is equated to a vector on the right-hand side.

Should one take the norm of 'u_f x u_{s,t}' ?

Thank you for spotting this typo. We fixed it!

13.

I would have thought that the length of the sperm flagellum is tightly controlled.

What evidence is there for the assummed variability of flagella length?

(Judging this variability from microscope images can be difficult because the distal tip of flagella often is difficult to track.)

The numbers are based on a citation [49] (M Cummins and P F Woodall. On mammalian sperm dimensions.Journal of reproduction and fertility, 75(1):153–175, sep 1985. ISSN 1470-1626. doi:10.1530/jrf.0.0750153) which states lengths between 50 and 80 µm. A normal distribution with 65 mean and 5 std ensures via the 3 sigma rule that 99,7% of sperm lengths are within the given range.

14.

The authors state themselves that their simulation framework is extendable.

Did the authors consider making their simulation code freely available as open source?

The complete Python model code is available in a public git repository at

https://ford.biologie.hu-berlin.de/jorin/female_sperm_selection

under the GNU *General Public License*, version *3*. The repository contains all files necessary to simulate the model and reproduce the presented figures. Additionally, a yml file is provided to set up a conda virtual environment to avoid complications due to package dependencies when running the model.

15.

For the rheotaxis model given in Eq. (S46), the rate of alignment is proportional to the exteral flow velocity (which is plausible),

but also proportional to the swimming speed of the sperm cell itself, which is less clear.

Can the authors motivate this modeling assumption better?

The assumption here is that the force responsible for turning the sperm is the shear stress, which depends on the flow velocity along the sperms boundary. This flow velocity should depends on the fluid velocity as well as the sperm velocity. However, we agree that a more precise dependency should implemented.

Minor comments

- The authors may want to consider to add a "z-axis" to Fig. 1B.

We considered the reviewers suggestion, but as there is a z-axis in Figure 3, we would like to keep Figure 1 as it is for esthetic reasons.

- The authors sometimes denote the time step sometimes by 'delta t', sometimes by 'dt' : I assume these are the same?

We replaced all δt and dt by Δt as those are discrete timesteps.

- Page 3: While introducing the maximal flow velocity '50 um/s', the authors may already quote the measurement from Miki&Clapham, to inform the reader about the expected order of magnitude.

- Figure 2DEF: add scale bar? add label 'end point' at black dots?

We added scale bars and labels to Figure 2DEF. Simulation times equaled 30 seconds for all figures.

- Reference 7: journal missing?

We included the missing journal:

S.S. Suarez, A. A. Pacey Sperm transport in the female reproductive tract

Human Reproduction Update, Volume 12, Issue 1, January/February 2006, Pages 23–37, https://doi.org/10.1093/humupd/dmi047

- Page 14: Isn't Eq. (S40) just the same as Eq. (S39) with 'd_max=r_com' and 'd=d_max-r' ?

Yes, it is the same. The difference is that Eq S39 was used for solely radial compartments and is calculated analytically, while Eq. 40 uses apriori calculated distance arrays which store the distance to the wall.

- I appreciated Fig. S4 but think it could be extended.

For example, to be better able to follow the text on the "ellipsoidal sensing zone" on page 12, an illustration similar to Fig. S4B would be very useful.

- Supplementary Note 2A:

I found the cross-references to the corresponding Figure S4 rather sparse. It would help the reader if in each paragraph reference to the corresponding panel of Fig. S4 would be made.

We improved the cross referencing in **Supplementary Note 2A**.

- References should be provided for the pusher-type swimming of sperm cells.

As reference for the pusher type swimming of sperm we added the following publication:

Jens Elgeti and Gerhard Gompper. Microswimmers near surfaces.European PhysicalJournal: Special Topics, 225(11-12):2333–2352, nov 2016. ISSN 19516401. doi:10.1140/epjst/e2016-60070- 6.

In section **Box model reproduces *in vitro* dynamics of sperm**.:

Hydrodynamically, sperm cells are pushers (29), ...

<u>Reviewer #3:</u> The paper entitled, "Sperm migration in the genital tract - in silico experiments identify key factors for reproductive success" describes an agent-based simulation of sperm migration through the female reproductive tract. Like previous work published in literature, the main focus of the paper is the migratory characteristics of the spermatozoa and the impact on the outcome which is the number of spermatozoa reaching the oviduct.

Generally I like the paper. It shows a simple approach and a very simple strategy to model the sperm migration in the female reproductive tract. Maybe the most important feature of this paper is the inclusion of the potential impact of the immune system in the spermatozoa transport within the female reproductive tract and how it can affect the number of spermatozoa reaching the site of the fertilization. The other interesting aspect of the paper is the author's attempt to include a very simple approach for inclusion of fluidity dynamics in their calculations and in their model.

We thank the reviewer for his/her constructive evaluation of our work! We tried our best to address the following comments and hope that the revised version might be agreed to become published.

In my opinion the same issue of simplicity is probably also the main weakness of this paper. The authors have reconstructed the 3D geometry of the tract, but using idealised functions to describe the different cross-sections, rather than medical images. There is no additional representation of internal geometry/folds as far as I can make out. This point needs to be discussed and needs to be mentioned as a limitation of the current paper.

We agree with the reviewer. The geometric properties, particularly of the cervical compartment are clearly simplified. This is undoubtedly a weakness of the model, however, we would like to emphasize that the model with a principle but simplified inclusion of folds predicts the physical process of thigmotaxis as determinant of agent's success. To what extent the more realistic geometric peculiarities interfere with the principle of wall alignment remains a topic of extension.

We included the following sentence in the **Discussion** section to comment the simplified reconstruction of the native situation:

"However, one should be cautious to compare total numbers for mainly three reasons: First, the immune system was modeled solely as a sperm removing process, which is drastically simplified. Second, idealized functions were used to simulate the much more complex architecture of genital tract compartments. Third, the deflection angles of sperm were drawn from normal distributions with standard deviations between 1 and 119 degree, which resulted in mean agent straightness values similar to the ones measured by Tung et al.(19)."

The model incorporates the vagina to the UTJ, but not the oviduct itself. They consider the effects of fluid/mucus flow, but only as a persistent velocity vector that is taken into account when calculating sperm direction, not anything more sophisticated. The sperm behaviour is represented as a simple motion, without consideration of many other aspects of spermatozoa physiology, for example capacitation, hyperactivated motility is included. Also, no consideration of underlying processes in the female reproductive tract e.g. the effect of the reproductive cycle, hormones and even the spatial topography of the reproductive tract in the cow. These points are hardly mentioned in the discussion of the paper. The reader needs to be informed of all the limitations of the paper and it needs extensive discussion.

The above mentioned points match the suggestions of reviewer 2.

Indeed, there exists a bunch of biophysical and biochemical processes to become considered in a model. However, we did not yet intend to include the sperm behaviour in the oviduct in the presented study because it is still and first a challenge in many mammalian species to understand reproductive failures of sperms on their way to the oviduct. This is particularly true if artificial insemination is needed as it is in human and endangered animals or animal breeding.

To give an example for future models, we could imagine a more sophisticated description of fluid sperm interactions to mimic cyclic changes in the female genital tract OR a change in sperm rheotaxis parameters, i.e. introducing faster alignment if one assumes less viscous fluid and slower alignment for more viscous fluids.

The many aspects dedicated to capacitation sperm binding to oviduct epithelial cells and removal by hypercativation occur in the near vicinity of the egg. Therefore, processes such as chemotaxis or thermotaxis are investigated and discussed in the literature. However, we think that a review on chemokinesis, chemotaxis, thermotaxis would be beyond the scope of the study. Therefore we decided to restrict this part of outlook to the keywords, which are surely familiar to the more specialized reader.

However, we extended the **Discussion** in the required direction:

"The complex processes occurring to sperm in the oviduct are a further topic to extend/complete the simulation of sperm migration to the oocyte. Chemotaxis, thermotaxis, and rheotaxis as well as sperm interactions with epithelial cells and capacitation-related metabolic changes leading to hyperactivation of sperm motion are currently regarded and investigated as key processes prior to fertilization (13, 33, 43)."

I think the paper would be improved if authors present a more comprehensive account of other papers in the field and in particular mentioning the other models produced in other species. A more in depth comparison with their approach would be very useful. In addition it will be very important to discuss and mention the shortcomings and limitations of their approach and also the future general avenues for further improvement of modelling spermatozoa migratory behaviour in the female reproductive tract.

Again this request matches the critical points of reviewer 2.

Therefore, we performed several further simulations to compare the outcome of our model to existing models and experimental approaches on sperm motion. Here we repeat the answers to reviewer 2.

In order to justify our minimal models, we compared our model of thigmotaxis with data published by Nosrati et al. (2016) (https://www.nature.com/articles/srep26669), while the rheotaxis model was compared to data and model from Kantsler et al. (2014) (https://elifesciences.org/articles/02403).

Thigmotaxis:

Nosrati et al. recorded sperm x and y positions in channels with different widths. They found an increased sperm concentration near walls and in channel corners. Depending on this channel width different percentages of corner, wall and bulk swimmers were identified. Our model reproduces the accumulation of sperms in corners and at walls. The results are included in

Figure 2G and H. As the percentage of corner and wall swimmers are slightly underestimated, the effect of thigmotaxis in our model is likely also underestimated.

We included the following statement in the section **Box model reproduces *in vitro* dynamics of sperm:**

"Nosrati et al. (31) measured bull sperm densities in channels of varying width (50µm,100µm and 400µm). Depending on their distance to channel wall and corners sperms were classified as wall, corner or bulk swimmer. With increasing channel size the percentage of corner swimmers decreased from approximately 80% to around 30%. The presented model predicts a decrease from approximately 60% to around 10% (Fig. 2G, H). Hydrodynamic properties determining motion and orientation of sperms at surfaces are addressed in more advanced models (29, 32), while our minimal model is based on vector additions when agents are in proximity of the compartment boundary. In contrast to the advanced models our sperms are not attracted towards the wall over long distances.This potentially leads to the slightly lower percentage of agents classified as corner and wall swimmers Fig. 2G and H, compared with data from Nosrati et al. (31). As a result, the effect of thigmotaxis is likely underestimated. However,the agents tend to swim along surfaces as shown in Fig. 2E."

Comparing the thigmotaxis model with data from Nosrati et al. shows that we slightly underestimate this effect. While Nosrati et al. report that 80 % of sperms are corner swimmers in a channel with 50 µm width, our model predicts around 60 %. This is most likely due to missing long distance attraction between boundaries and sperms, which is included in more detailed models for thigmotaxis (Elgeti & Gompper 2016, https://link.springer.com/article/10.1140/epjst/e2016-60070-6).

Rheotaxis:

Kantsler et al. (2014) tracked bull sperm positions in a microfluidic device during flow reversal. Further they present a sophisticated model of sperm rheotaxis which includes fluid viscosity. A feature our description is missing. Depending on the fluid viscosity Kantsler er al. measured a reorientation time of 5s (at 1mPas) to 50s (at 12 mPas, which is roughly 4x higher than the viscosity of natural ejaculate). Our model predicts a reorientation time around 20s and thereby perfectly agrees with the data from Kantsler et al. The results are included in Figure 2I.

We included the following statement in the section **Box model reproduces *in vitro* dynamics of sperm:**

"To justify the outcome of our minimal model in comparison with a more detailed model by Kantsler et al. (23), we tested the behavior of our agents upon fluid flow reversal. In agreement with the referred study, after an initial peak in the upstream velocity, agents re-orientate within 20s (Fig. 2I)"

We wish to thank you again for the option to submit a revised version of our manuscript and would be very happy if the revised manuscript would now be suitable for publication.

Sincerely,

Edda Klipp
